# Overlapping Patterns of Gene Expression Between Gametophyte and Sporophyte Phases in the Fern *Polypodium amorphum* (Polypodiales)

**DOI:** 10.3389/fpls.2018.01450

**Published:** 2018-10-09

**Authors:** Erin M. Sigel, Eric Schuettpelz, Kathleen M. Pryer, Joshua P. Der

**Affiliations:** ^1^Department of Biology, University of Louisiana at Lafayette, Lafayette, LA, United States; ^2^Department of Botany, National Museum of Natural History, Smithsonian Institution, Washington, DC, United States; ^3^Department of Biology, Duke University, Durham, NC, United States; ^4^Department of Biological Science, California State University Fullerton, Fullerton, CA, United States

**Keywords:** gametophyte, life cycle, MADS-box, phototropins, sporophyte, terpene synthases, transcriptomics

## Abstract

Ferns are unique among land plants in having sporophyte and gametophyte phases that are both free living and fully independent. Here, we examine patterns of sporophytic and gametophytic gene expression in the fern *Polypodium amorphum*, a member of the homosporous polypod lineage that comprises 80% of extant fern diversity, to assess how expression of a common genome is partitioned between two morphologically, ecologically, and nutritionally independent phases. Using RNA-sequencing, we generated transcriptome profiles for three replicates of paired samples of sporophyte leaf tissue and whole gametophytes to identify genes with significant differences in expression between the two phases. We found a nearly 90% overlap in the identity and expression levels of the genes expressed in both sporophytes and gametophytes, with less than 3% of genes uniquely expressed in either phase. We compare our results to those from similar studies to establish how phase-specific gene expression varies among major land plant lineages. Notably, despite having greater similarity in the identity of gene families shared between *P. amorphum* and angiosperms, *P. amorphum* has phase-specific gene expression profiles that are more like bryophytes and lycophytes than seed plants. Our findings suggest that shared patterns of phase-specific gene expression among seed-free plants likely reflect having relatively large, photosynthetic gametophytes (compared to the gametophytes of seed plants that are highly reduced). Phylogenetic analyses were used to further investigate the evolution of phase-specific expression for the phototropin, terpene synthase, and MADS-box gene families.

## Introduction

**FIGURE 1:**
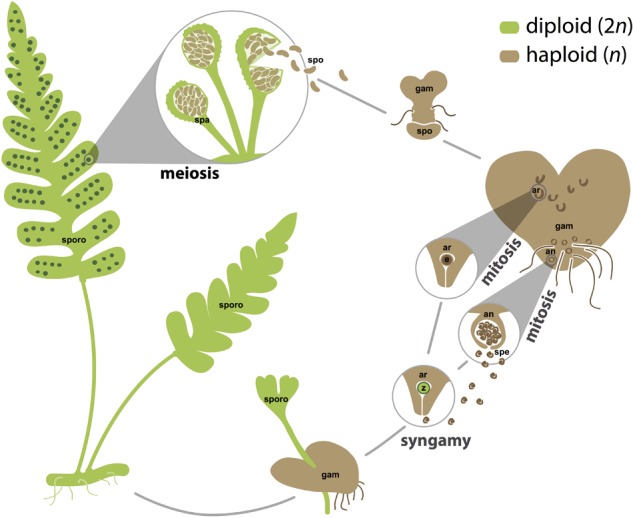
The homosporous fern life cycle. Sporophyte tissues, which are usually but not necessarily diploid, are shown in green. Gametophyte tissues and spores, which are often but not necessarily haploid, are shown in brown. Spores are generated by meiosis in sporangia. Gametes, both eggs and sperm, are generated by mitosis in archegonia and antheridia, respectively. For simplicity, fertilization is depicted between an egg and sperm from the same gametophyte, but fertilization is also likely to occur between gametes from different gametophytes that are derived from the same or different sporophytes. an, antheridium; ar, archegonium; e, egg; gam, gametophyte; spa, sporangium; spe, sperm; spo, spore; sporo, sporophyte; z, zygote. Images are not to scale.

**FIGURE 2:**
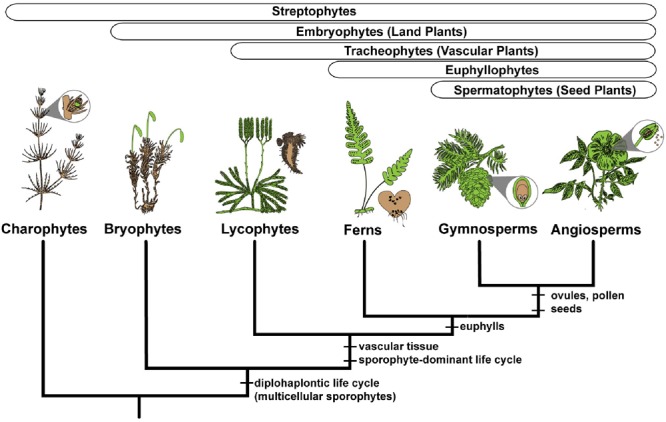
Simplified phylogeny of the major clades of streptophyte plants, illustrating the gametophyte (colored brown) and sporophyte (colored green) phases for exemplar lineages. Charophyte algae have a multicellular gametophyte and a single celled sporophyte. All embryophytes, or land plants, have multicellular gametophytes and multicellular sporophytes. Synapomorphies are shown for the major clades.

All land plants share a life cycle that alternates between a multicellular spore-producing phase (sporophyte) and a multicellular gamete-producing phase (gametophyte). However, it is in ferns that the manifestation of these two phases (also referred to as generations) as independent entities is most extreme (Niklas and Kutschera, 2010; Haufler et al., 2016). Fern sporophytes are typically long-lived, complex plants composed of roots, stems, and leaves (**Figure 1**). Fern gametophytes are considerably smaller (usually less than one centimeter long), possess fewer tissue types, and are often ephemeral, but are usually photosynthetic, and can sometimes persist independently for multiple growing seasons (Foster and Gifford, 1974; Sato, 1982; Raghavan, 2005; Watkins et al., 2007; Haufler et al., 2016; Pinson et al., 2017). Thus, fern sporophytes and gametophytes are distinct and separate organisms, varying in morphology, physiology, persistence, ecology, and usually ploidy, while sharing a common genome (Qui et al., 2012). The fern life cycle, with its two free-living phases, occupies a pivotal phylogenetic position in the spectrum of green plant life cycles, which ranges from the charophyte algae (i.e., the closest relative to land plants) life cycle that lacks a multicellular sporophyte phase to the angiosperm life cycle in which gametophytes have been reduced to a few cells (**Figure 2**; Kenrick and Crane, 1997a,b; Graham et al., 2000). Ferns, with their two free-living phases, represent an ideal but under-utilized resource for addressing questions pertaining to the morphological and functional differences between generations in life cycle evolution (Whittier, 1971; Barker and Wolf, 2010; Der et al., 2011).

The phase-specific morphologies and functions of fern gametophytes and sporophytes, like those of all land plants, result largely from different gene expression patterns (Graham et al., 2000), with some genes uniquely expressed in each phase. Previous isozyme, microarray, and RNA-Seq transcriptome profiling studies comparing gene expression between sporophytes and gametophytes in angiosperms and bryophytes uncovered a negative relationship between the morphological and functional divergence of the two phases and the similarity of their gene expression profiles (e.g., Tanksley et al., 1981; Gorla et al., 1986; Pedersen et al., 1987; Becker et al., 2003; Honys and Twell, 2003; Pina et al., 2005; Szövényi et al., 2011; Rutley and Twell, 2015). In general, the highly reduced male and female gametophytes of seed plants (the sister lineage to ferns) have reduced transcriptome profiles, expressing fewer and different genes than sporophyte tissues (Chettoor et al., 2014; Rutley and Twell, 2015). For example, in *Arabidopsis thaliana*, approximately one third of the genes expressed in sporophyte tissue are expressed in pollen, with approximately 10% of pollen-expressed genes being unique to that phase (Becker et al., 2003; Honys and Twell, 2003; Pina et al., 2005). In contrast, in the bryophyte *Physcomitrella patens* approximately 85% of genes are expressed by both the sporophyte and gametophyte (Ortiz-Ramírez et al., 2016). Similarly, a transcriptomic survey of *Lygodium japonicum* suggested that ferns may have more genes expressed in both gametophytes and sporophytes than do angiosperms (Aya et al., 2015).

Here, we evaluate the partitioning of gene expression between gametophyte and sporophyte phases in the well-studied, homosporous fern species *Polypodium amorphum* Sukds. This diploid member of the *Polypodium vulgare* species complex (Sigel et al., 2014) belongs to the leptosporangiate order Polypodiales (henceforth, polypod ferns) that encompasses approximately 80% of extant fern species (PPG I, 2016). *Polypodium amorphum* embodies the cytological, morphological, and life history traits typical of the clear majority of ferns. It has a high base chromosome number (*x* = 37; Haufler et al., 1993), a large genome size (C-value = 11.5 pg; Murray, 1985), and perennial sporophytes whose spores produce cordate, photosynthetic gametophytes. We generated sporophyte-leaf and whole-gametophyte transcriptomes from three individuals of *P. amorphum* (each collected from independent populations) to characterize expression profiles for this iconic polypod fern and to investigate its phase-specific gene expression in the broader context land plant life-cycle diversity. Despite having greater overlap in gene-family identity with seed plants, we found *P. amorphum* to have phase-specific expression profiles like those of its more distant relatives—bryophytes and lycophytes. Our study supports the hypothesis that plants with relatively large, photosynthetic gametophytes will exhibit substantially more overlap in the identity and expression levels of genes in their gametophyte and sporophyte phases regardless of their phylogenetic relatedness to each other or whether they have gametophyte-dominant or sporophyte-dominant life cycles. In addition, we investigate the phototropin, terpene synthase, and Type II MADS-box gene families to determine whether phase-specific gene expression can inform our understanding of gene family function and evolution.

## Materials and Methods

### Plant Material

We sampled sporophyte leaf material and whole gametophytes from three individuals of *P. amorphum* Suksd. (**Supplementary Table S1**). Species determination followed Haufler et al. (1993) and Sigel et al. (2014). Living sporophytes were initially collected from wild populations and transported to the glasshouses at Duke University, Durham, NC, United States, where rhizomes were cleaned to remove soil and repotted in Farfard Mix 2 (Sun Gro Horticulture Canada Ltd., United States). Plants were maintained under common glasshouse conditions (photoperiod 18 h: 6 h, light: dark, with luminosity of 200–400 Umol sec^-1^ cm^2^; 27–67% humidity; daytime temperature: 18.3–21.1°C; nighttime temperature: 17.8–20.6°C) for a minimum of 18 months prior to sampling for RNA extraction. Material from a single leaf was taken from each individual and flash frozen in liquid nitrogen 21 days after it had fully unfurled but before sporangia had developed.

Spores from each sporophyte individual of *P. amorphum* were collected and cleaned with bleach solution, as described in Hoshizaki (1975). For each individual, four replicated cultures of gametophytes were grown from spores in a modified Knop’s liquid medium to which 0.1% glucose was added (Miller and Miller, 1961; Smith and Robinson, 1969) under controlled conditions (photoperiod 12 h: 12 h, light: dark; light source: Philips 392282 40W Plant and Aquarium linear fluorescent bulb). At 55 days, a subset of each gametophyte culture was visually inspected under 100 × magnification to confirm the absence of gametangia. The gametophyte replicates for each individual, representing a range of pre-gametangial developmental stages, were then combined and flash frozen in liquid nitrogen. Leaf material and gametophyte samples were maintained at -80°C until RNA extraction.

### RNA Extraction, cDNA Library Construction, and Sequencing

For each of the three collections of *P. amorphum*, two separate RNA extractions were performed—one from 70 to 100 mg of sporophyte leaf material and one from 50 mg of whole gametophytes—using the Spectrum Plant Total RNA kit (Sigma-Aldrich, United States). A total of six mRNA libraries were constructed using the TruSeq RNA Sample Prep Kit (Illumina, United States), with the protocol modified to produce indexed, strand-specific, 100-bp paired-end libraries following Borodina et al. (2011). Equimolar amounts of the libraries were pooled prior to sequencing on two lanes of Illumina HiSeq 2000 (Illumina, United States) at the Duke Center for Genomic and Computation Biology. After sequencing, reads from each library were sorted by index, and the number and quality of reads from each sample were evaluated with FastQC v. 3-5-2012 (Andrews, 2010). Adapter sequences and low-quality reads were removed using Trimmomatic v. 0.30 (parameters: PE - ILLUMINACLIP:TruSeq3-PE.fa:2:30:10:8:true HEADCROP:10 TRAILING:20 SLIDINGWINDOW:10:30 MINLEN:30; Bolger et al., 2014). All reads that passed a second round of quality filtering using FastQC v. 3-5-2012 (Andrews, 2010) were used in *de novo* transcriptome assembly and gene expression analyses.

### *De novo* Reference Transcriptome Assembly and Annotation

Because there are no published genomes of polypod ferns, we constructed a *de novo* reference transcriptome for *P. amorphum* from the filtered, paired-end reads generated from all six cDNA libraries using Trinity v. r20140413 (Grabherr et al., 2011) on the Duke Shared Cluster Resource. A strand-specific protocol (*RF*) was used with a fixed k-mer size of 25 and a maximum insert size of 800 bp. All reads were then mapped back to the initial reference transcriptome using Bowtie v. 1.0.1 (Langmead et al., 2009) and quantified with RSEM v. 1.2.14 (Li and Dewey, 2011) using the wrapper script align_and_estimate_abundance.pl (Grabherr et al., 2011). Transcripts (i.e., gene isoforms) representing less than 1% of the per gene (i.e., subcomponent in Trinity) expression level were discarded as possible artifacts of the transcriptome assembly algorithm (Grabherr et al., 2011; Haas et al., 2013).

To restrict differential gene expression analyses to protein coding genes, we translated transcripts using ESTScan, a program that detects coding regions and corrects sequencing errors that cause frameshifts (Iseli et al., 1999). To identify and remove non-plant contaminant sequences, we queried the resulting protein sequences against the RefSeq non-redundant peptide database (nr; release 09/2015; O’Leary et al., 2016) using BLASTP v. 2.2.30 (e-value:1e-5, max_target_seqs: 5; Camacho et al., 2009). General taxonomic information (bacteria, fungi, *Homo sapiens*, Viridiplantae, other Metazoan, virus, and other) was retrieved for the five best subject sequences for each reference transcript from the NCBI Taxonomy database (Federhen, 2012). A transcript was identified as a non-contaminant (i.e., plant) if the best subject sequence and ≥ 50% of the best five subject sequences belonged to Viridiplantae. Transcripts with hits to nr but not meeting these criteria were identified as contaminants. Transcripts without hits to nr were designated as “no hit,” representing potential fern-specific genes. Non-contaminant and “no hit” sequences were included in the final reference transcriptome that was used for subsequent differential-expression (DE) analyses. The completeness of the final *P. amorphum* transcriptome assembly was assessed by quantifying the presence of universal single copy orthologs using BUSCO software and the Embryophyta odb09 dataset under the trans setting (Simão et al., 2015). For comparative purposes, a previous published transcriptome of *L. japonicum* gametophyte and sporophyte tissue (Aya et al., 2015) was also subjected to BUSCO analysis.

The final *P. amorphum* reference transcriptome was annotated to orthogroup (i.e., narrowly defined gene lineage) using the global PlantTribes gene family classification (Wall et al., 2008). Briefly, a Markov Cluster algorithm as implemented in OrthoMCL (Li et al., 2003) was used to cluster transcripts with all predicted protein coding sequences from 22 land plant genomes, representing bryophytes (*P. patens*), lycophytes (*Selaginella moellendorffii*), and a diversity of angiosperms (including *Amborella trichopoda, Oryza sativa*, and *A. thaliana*). Functional annotations for each transcript were obtained by cross referencing its assigned orthogroup number with a previously compiled set of TAIR (Berardini et al., 2015), PFAM (Finn et al., 2016), and Gene Ontology (GO) annotation terms (The Gene Ontology Consortium, 2015).

### Quantification and Functional Classification of Phase-Specific Gene Expression Profiles

For each of the six *P. amorphum* libraries (three sporophyte, three gametophyte), all reads that mapped to transcripts identified as contaminant sequences were removed from the read files. All remaining reads were remapped to the final reference transcriptome using Bowtie v. 1.0.1 (Langmead et al., 2009) as implemented through the Trinity package v. r20140413 (Grabherr et al., 2011) using parameters specifically for paired-end, strand-specific reads (Haas et al., 2013). Maximum likelihood (ML) read abundances were calculated for each gene using RSEM v. 1.2.13 (Li and Dewey, 2011). The Trinity package script abundance_estimates_to_matrix.pl (Haas et al., 2013) was used to generate a matrix of read abundances of all genes for the six libraries. This matrix was filtered to exclude genes that were very weakly expressed (i.e., not represented by at least ten reads in at least three libraries).

Differential expression (DE) analyses among samples were performed using the DESeq2 package in R v. 3.1.0 (Love et al., 2014; R Core Team, 2015), and incorporated a multifactor block design to account for the paired sporophyte and gametophyte samples (∼ phase + individual). The BH method was used to adjust *p*-values to account for false discovery of DE genes (Benjamini and Hochberg, 1995; Robinson and Oshlack, 2010). The number of DE genes between the phases was assessed at adjusted *p*-values (padj) of 0.1, 0.02, 0.01, 0.002 and 0.001, and at log2-fold change (FC) in expression of 2, 4, and 6. DE genes were assigned to one of four categories: gametophyte-specific; gametophyte up-regulated (i.e., more highly expressed in the gametophyte than in the sporophyte); sporophyte-specific; or sporophyte up-regulated (i.e., more highly expressed in the sporophyte than in the gametophyte). A gene was identified as gametophyte-specific if it met the padj and FC threshold criteria and had a mean normalized count of < 1.0 for the three sporophyte samples. Similarly, a gene was determined to be sporophyte-specific if it met the padj and FC threshold criteria and had a mean normalized count of < 1.0 for the three gametophyte samples.

DE genes having padj ≤ 0.002, log2FC ≥ 2, and associated GO terms were subjected to GO enrichment analyses using a Fisher’s exact test as implemented in goatools^1^. GO terms associated with genes belonging to the four aforementioned categories of DE were compared to GO terms for all genes. A GO term was determined to be under- or over-represented using a false discovery rate *p*-value threshold (FDR) of 0.05. Fold enrichment was calculated for each gene by dividing the proportion of DE genes assigned a particular GO term by the proportion of all genes assigned to that GO term. Fold enrichment values > 1 indicate GO terms that are enriched or over-represented. Fold enrichment values < 1 indicate GO terms that are purified or under-represented.

### Phylogenetic Analyses of Phase-Specific Expression for Exemplar Gene Families

To assess phase-specific gene expression in the context of enriched GO terms and orthogroup evolution across land plants, three gene families—phototropin, terpene synthases (TPS), and Type II MADS-box—were selected for further phylogenetic analyses. The annotated *P. amorphum* reference transcriptome was mined for transcripts assigned to orthogroups for each gene family (see **Supplementary Table S2**; phototropin: orthogroup 571; terpene synthase: orthogroups 94, 716, 3728, 14080, 27466; Type II MADS-box: orthogroups 278, 1109, 1536, 2959, 3518, 4128, 12048). Prior to phylogenetic analyses, *P. amorphum* transcripts for each gene family were aligned with transcripts and genomic sequences from preexisting publications and public databases (phototropin: all sequences from Li et al., 2015; terpene synthase: 184 plant, microbial, and fungal TPS sequences downloaded from the Pfam database, Finn et al., 2016; Type II MADS-box: sequences from Kofuji and Yamaguchi, 1997; Münster et al., 1997, 2002; Hasebe et al., 1998; Kwantes et al., 2011; Gramzow and Theissen, 2013; Huang et al., 2014), as well as with transcripts from the *Lygodium japonicum* Transcriptome Database (Aya et al., 2015). A nucleotide alignment for the phototropin dataset and amino acid alignments for the TPS and Type II MADS-box datasets were compiled in Mesquite v. 3.10 (Maddison and Maddison, 2016). For the phototropin alignment, indels resulting from the addition of *P. amorphum* and *L. japonicum* transcripts, presumably corresponding to unalignable regions excluded from the published alignment of Li et al. (2015), were removed. For the TPS alignment, only the conserved C-terminal domain was included in the final alignment to facilitate the analysis of TPS sequences across disparate taxonomic groups. Similarly, alignment of the Type II MADS-box sequences was restricted to the relatively conserved SRF-like MADS-box and K-box domains.

PartitionFinder v. 1.0.1 (Lanfear et al., 2012) was used to determine the appropriate model of nucleotide evolution for the phototropin alignment (partitioned by codon position with the GTR + I + G model independently applied to each position). PartitionFinderProtein v. 1.0.1 (Lanfear et al., 2012) was used to identify the optimal model of peptide evolution for the TPS alignment (VTF) and the Type II MADS-box alignment (LG + G models for the SRF-like MADs-box domain and the JTT + G model for the K-box domain).

For each gene family alignment, a maximum likelihood (ML) tree search was conducted using RAxML (Stamatakis, 2006) with eight independent runs from different random-addition starting trees. Node support was assessed with 1000 bootstrap replicates. MrBayes v. 3.2.5 (Ronquist et al., 2012) was used for Bayesian inference (BI), with four MCMC runs of four chains each and trees sampled every 1000 generations for a total of 10 million generations. The same partitions and evolutionary models as for the ML analysis were employed, with unlinked substitution parameters and the rate priors varying among subsets. Tracer v. 1.6 (Rambaut et al., 2014) was used to check chain convergence and proper mixing. For each MCMC run, 2500 trees were discarded as burn-in. All model assessment and phylogenetic analyses were conducted on the Smithsonian Institution High Performance Cluster (SI/HPC). Alignments and tree files are deposited in Figshare (doi: 10.6084/m9.figshare.c.422914).

## Results

### The *Polypodium amorphum* Transcriptome

After sequencing and quality filtering, 41–61 million read pairs and 4–6 million single-end reads were obtained for each of the six transcriptome libraries (**Supplementary Table S3**), with all reads for the six libraries used to construct the *de novo* reference transcriptome. Following filtering to remove assembly artifacts, duplicate transcripts, and non-coding and contaminant sequences, assembly resulted in a final reference transcriptome of 150,141 transcripts corresponding to 95,755 genes with an N50 of 1377 bases (**Supplementary Tables S4, S5**; doi: 10.6084/m9.figshare.c.4229141). The clear majority of genes, 75,480 (78.8%), are represented by one assembled transcript (**Supplementary Figure S1**). Raw Illumina reads are deposited in NCBI’s BioProject database ^2^ (BioProject: PRJNA341394, BioSamples: SAMN05721156, SAMN05721260–2, SAMN05721283–4). BUSCO assessment indicates that 71.4% of universal single-copy Embryophyta orthologs are represented as complete sequences, with another 3.1% represented as partial sequences in the *P. amorphum* transcriptome (**Supplementary Figure S2**). By comparison, 53.6% of universal single-copy orthologs are represented as complete sequences and 7.2% are represented as partial sequences in the *L. japonicum* transcriptome. To the best of our knowledge, the *P. amorphum* transcriptome is the most complete transcriptome assembly of combined fern gametophyte and sporophyte tissue to date.

**FIGURE 3:**
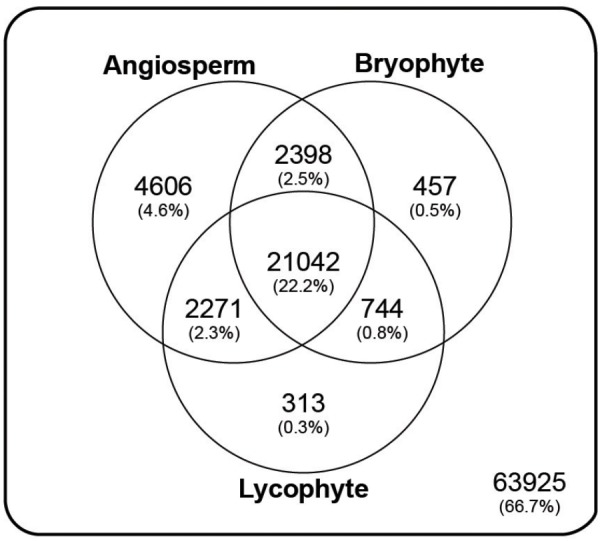
Taxonomic summary of the annotated *Polypodium amorphum* reference transcriptome. Of the 98000 total genes, 63925 were not assigned to an orthogroup in the Plant Tribes classification (Wall et al., 2008). All remaining genes were assigned to an orthogroup with a predicted protein in the genome of at least one angiosperm species, the lycophyte *Selaginella moellendorffii*, and/or the bryophyte *Physcomitrella patens*.

Annotation resulted in 62,305 (41.5%) transcripts, representing 31,831 (33.2%) genes, with significant similarity (e-value < 1e^-8^) to at least one of 10,292 orthogroup HMM profiles present in the PlantTribes classification (**Figure 3** and **Supplementary Figure S3**; Wall et al., 2008). A summary of orthogroup numbers and annotation terms associated with *P. amorphum* transcripts is provided in **Supplementary Table S2**.

### Identification and Functional Classification of Phase-Specific Gene Expression Profiles

**FIGURE 4:**
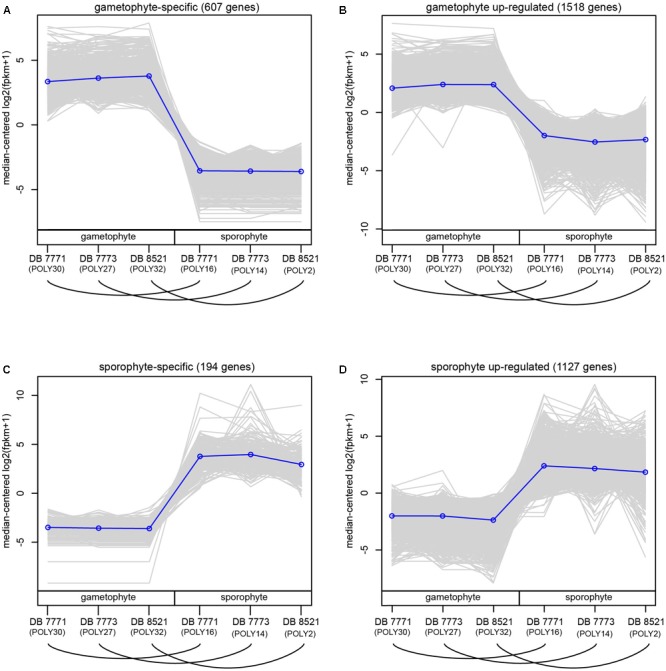
Normalized expression (log2-transformed FPKM + 1) of genes assigned to four categories of differential expression using the criteria of log2FC ≥ 2 and padj ≤ 0.002. Each gray line represents the expression level of a particular gene relative to the median expression level of all genes in that expression category. Blue lines represent the median expression level for each expression category. Samples are labeled as gametophyte or sporophyte, with an arched line connecting samples derived from the same *Polypodium amorphum* individual. **(A)** gametophyte-specific genes; **(B)** gametophyte up-regulated genes; **(C)** sporophyte-specific genes; **(D)** sporophyte up-regulated genes.

For each sample, 20–31 million reads were mapped back to the final reference transcriptome (**Supplementary Table S6**). After filtering to exclude very weakly expressed genes, 35,169 genes (including both those with and without annotation to the Plant Tribes orthogroup classification) were retained for the DE analyses (**Supplementary Table S7**). Using thresholds of FC ≥ 2 and a padj < 0.002, 3447 genes (9.8%) were supported as differentially expressed between sporophyte leaf tissue and whole gametophytes. These thresholds were selected to encompass the mean FC value (2.86) and minimize false detection, thereby providing a conservative estimate of differentially expressed genes (**Supplementary Figures S4–S6**), which were divided into four groups: 607 genes (17.6%) are gametophyte-specific; 1518 (44.0%) are up-regulated in the gametophyte; 194 (5.6%) are sporophyte-specific; and 1128 genes (32.7%) are up-regulated in the sporophyte (**Figure 4** and **Supplementary Figure S6**).

**FIGURE 5:**
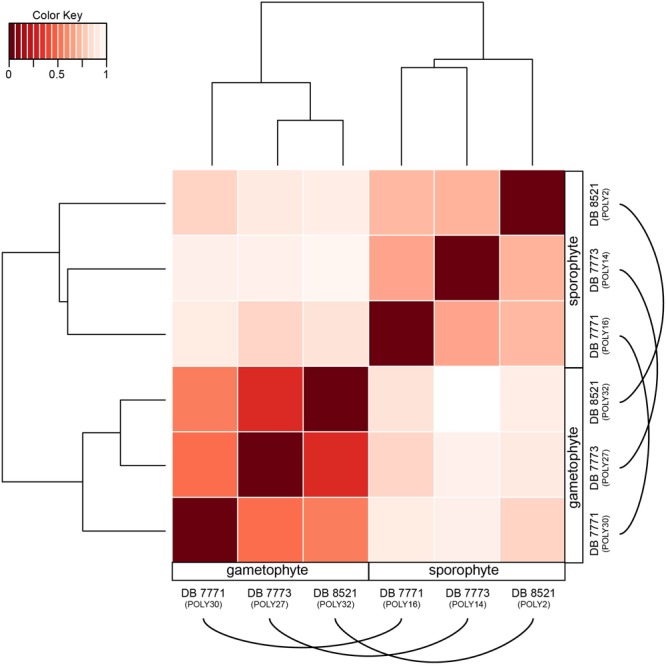
Heat map showing the hierarchically clustered Euclidean distance matrix resulting from comparing the transcript expression values (log2-transformed FPKM + 1) for each pair of *Polypodium amorphum* samples. Samples are labeled as gametophyte or sporophyte, with an arched line connecting samples derived from the same *P. amorphum* individual.

Despite a nearly 90% overlap in gene identity expressed in both the sporophyte and gametophyte phases of *P. amorphum*, each phase does have a distinctive transcription profile. This pattern is strikingly illustrated in **Figure 5**, which depicts the hierarchical clustering of samples by total expression values for all 35,169 genes (log2-normalized reads) and Euclidean distance of total gene expression between samples. Gametophyte samples and sporophyte samples were divided into two mutually exclusive groups due to gametophyte samples having more genes exhibiting extremely high and less variable gene expression than the sporophyte samples (**Figure 4**). Hence, global expression patterns—incorporating information from both gene identity and expression level—are more strongly dictated by phase rather than by the unique identity of the *P. amorphum* individual.

Of the genes included in the DE analysis, 18,886 genes had associated GO Gene Ontology (GO) terms and were used as the population gene set for GO enrichment analyses. Four sample gene sets, comprised of DE genes (FC ≥ 2, padj ≤ 0.002) with associated GO terms were used for enrichment analyses: 230 gametophyte-specific genes, 773 gametophyte up-regulated genes, 86 sporophyte-specific genes, and 669 sporophyte up-regulated genes. Using a FDR *p*-value threshold of 0.05, seven GO terms were identified as enriched or over-represented in gametophyte-specific genes and 105 GO terms were purified or under-represented (**Supplementary Table S8**). For the gametophyte up-regulated genes, 16 GO terms were enriched and 59 GO terms were under-represented. For the sporophyte-specific genes, no enriched GO terms were recovered, but 16 GO terms were under-represented. For the sporophyte-specific genes, 18 GO terms were enriched and 173 GO terms were under-represented. **Table 1** summarizes the most highly enriched GO terms in the four categories of differentially expressed genes.

**Table 1:** Summary of the most highly enriched Gene Ontology (GO) terms for categories of differentially expressed genes at a threshold of log2FC ≥ 2 and padj ≤ 0.002.

Enriched GO terms^†^	GO Term Level^∗^	Fold enrichment	FDR *p*-value
**Gametophyte-specific expression**		
** Cellular component**		
cell wall	2	7.465	0.004
**Molecular function**		
terpene synthase activity	5	11.096	0.024
catechol oxidase activity	5	21.289	0.000
acireductone dioxygenase [iron(II)-requiring] activity	5	27.371	0.004
**Expression up-regulated in gametophytes**		
** Biological processes**		
regulation of macromolecule metabolic process	4	1.631	0.008
regulation of cellular macromolecule biosynthetic process	6	1.661	0.004
regulation of nucleobase-containing compound metabolic process	5	1.658	0.004
microtubule-based process	3	3.998	0.000
microtubule-based movement	4	4.515	0.000
**Molecular function**		
microtubule motor activity	8	4.515	0.000
ribonucleoside-diphosphate reductase activity, thioredoxin disulfide as acceptor	5	13.573	0.008
**Expression up-regulated in sporophytes**		
** Biological processes**		
oligopeptide transport	5	6.327	0.000
carbohydrate metabolic process	6	3.148	0.000
**Cellular component**		
integral component of membrane	3	1.668	0.016
membrane	1	1.942	0.000
**Molecular function**		
catechol oxidase activity	5	7.319	0.010
malonyl-CoA decarboxylase activity	5	5.293	0.012
cysteine-type peptidase activity	5	3.471	0.010
oxidoreductase activity, acting on peroxide as acceptor	3	3.297	0.006
hydrolase activity, hydrolyzing O-glycosyl compounds	4	2.631	0.000
hydrolase activity, acting on glycosyl bonds	3	2.647	0.000
hydrolase activity, acting on ester bonds	3	1.913	0.002
hydrolase activity	2	1.587	0.000

*^†^There are no enriched GO terms for sporophyte-specific genes.*

*^∗^Highest level GO term associated with a particular set of differentially expressed genes.*

### Phylogenetic Analyses of Phase-Specific Expression for Exemplar Gene Families

Our phylogenetic reconstructions of the phototropin, terpene synthase, and Type II MADs-box gene families largely confirm previously published phylogenies. For each gene family, the identities of *P. amorphum* transcripts were putatively determined based on their inclusion in well-supported, previously characterized clades (e.g., seed-plant type terpene synthases; Li et al., 2012).

#### Phototropin

**FIGURE 6:**
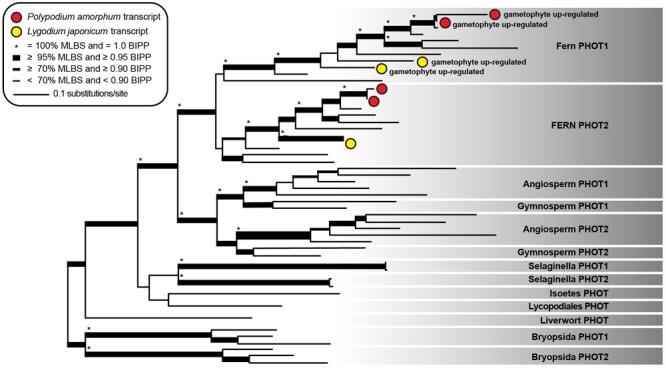
Phylogram of the best maximum likelihood tree of phototropin sequences. Shading indicates taxonomic specific phototropin clades as described in Li et al. (2015). Red and yellow circles represent single transcripts from the ferns *Polypodium amorphum* and *Lygodium japonicum*, respectively, that are up-regulated in the gametophyte phase. Node support values are as indicated in the inset legend. MLBS, maximum likelihood bootstrap value; BIPP, Bayesian inference posterior probability.

The topology and node support for our best ML tree (likelihood score = -47805.189182) was largely congruent with that of Li et al. (2015), with well-supported sister clades comprising fern sequences of phototropin 1 (fern PHOT1) and phototropin 2 (fern PHOT2; **Figure 6**). Two *P. amorphum* transcripts, putatively representing isoforms of a single gene, and two *L. japonicum* transcripts are well-supported as nested within the fern PHOT1 clade and are up-regulated in the gametophyte-phase. Similarly, two *P. amorphum* transcripts and one *L. japonicum* transcript are well-supported as nested within the fern PHOT2 clade.

#### Terpene Synthase

**FIGURE 7:**
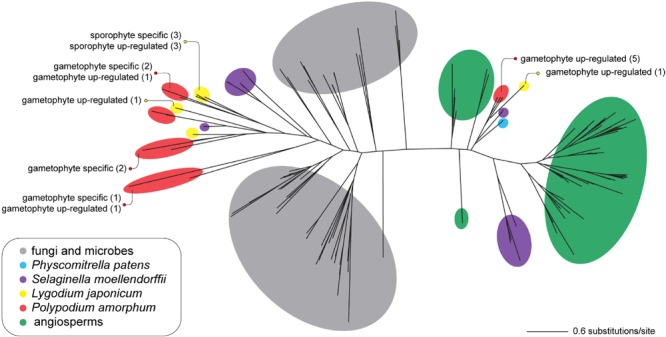
Unrooted phylogram of the best maximum likelihood tree of terpene synthase sequences. Colored circles indicate the taxonomic identity of particular sequences (see inset legend). Text describes the number of transcripts that are phase specific or up-regulated in the ferns *Polypodium amorphum* (red) and *Lygodium japonicum* (yellow). Node support values are provided at Figshare (doi: 10.6084/m9.figshare.c.4229141).

**FIGURE 8:**
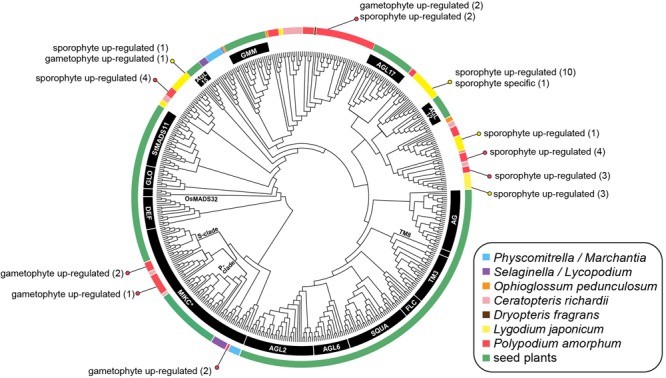
Cladogram of the best maximum likelihood tree of land plant Type II MADS-box sequences. Specific MADS-box genes, as described by Kwantes et al. (2011) and Gramzow and Theissen (2013), are indicated by black lines or along branches. Colored lines indicate the taxonomic identity of particular sequences (see inset legend). Colored dots and text describes the number of transcripts that are phase-specific or phase up-regulated in the ferns *Polypodium amorphum* (red) and *Lygodium japonicum* (yellow). Node support values are provided at Figshare (doi: 10.6084/m9.figshare.c.4229141).

Consistent with the results of Li et al. (2012), the topology for the best ML tree (likelihood score = -42727.713859) divides sequences into two large, well-supported clades—one comprising primarily of seed plant TPS sequences, and a second comprising primarily of microbial and fungal TPS (MTPSL) sequences (**Figure 7**). *Polypodium amorphum* and *L. japonicum* transcripts were nested within both clades, with most fern TPS sequences shown to be more closely related to sequences from the lycophyte *S. moellendorffii* than to sequences from seed plants, fungi, or microbes (**Figure 8**). These results suggest that both *P. amorphum* and *L. japonicum* have and express both plant-type and microbial/fungal-type TPS, many of which are specific to or up-regulated in the gametophyte phase.

#### MADS-Box Genes

The topology of our best ML tree (likelihood score = -46202.290775; **Figure 8**) is largely congruent with previously published Type II MADS-box gene phylogenies (Kwantes et al., 2011; Gramzow and Theissen, 2013), with non-fern sequences united into clades corresponding to 16 MIKC^∗^ and MIKC^C^ Type II MADS-box genes. Five gametophyte up-regulated transcripts of *P. amorphum* fern sequences were nested within the MIKC^∗^ clade, either as sister to sequences derived from the fern *Ceratopteris* or as sister to the moss *P. patens.* Most remaining *P. amorphum* and *L. japonicum* sequences were united in three clades dispersed among, but not closely related to, 15 clades of MIKC^C^ genes. Both *P. amorphum* and *L. japonicum* expressed numerous phase-specific or phase-upregulated MIKC^C^ transcripts.

## Discussion

### *Polypodium amorphum* Transcriptome Suggests That Polypod Ferns Show Greatest Overlap in Gene Identify With Angiosperms

Genomic resources for ferns have lagged far behind other plant lineages, but are currently experiencing a burst of expansion with the publication of numerous transcriptomes and the first genome assemblies (reviewed in Sessa and Der, 2016, as well as Matasci et al., 2014; Frank et al., 2015; Grusz et al., 2016; Tomei and Wolniak, 2016; Wang et al., 2016; Zhang et al., 2016; Grossman et al., 2017; You et al., 2017; Li et al., 2018). The *P. amorphum* reference transcriptome is a crucial addition because it includes biological replicates and marks the first survey of gene expression in both sporophyte and gametophyte tissue of a polypod fern, a group accounting for ca. 80% of extant fern diversity (The Pteridophyte Phylogeny Group, 2016). Such resources are critical to a full understanding of plant life cycle evolution, as well as to the study of gene family evolution and phylogenomics among ferns and across land plants.

Using the Plant Tribes classification (Wall et al., 2008), approximately 33% of the genes represented in the *P. amorphum* reference transcriptome can be assigned to gene families also present in the genomes of at least one angiosperm taxon, the lycophyte *S. moellendorffii*, and/or the bryophyte *P. patens*. Approximately 22% of the genes belong to families present in all three plant lineages (**Figure 3**). While these results suggest that most genes expressed in *P. amorphum* are poorly characterized and may be fern specific, they also broadly support the notion that gene family identity across all land plants is substantially conserved (Nishiyama et al., 2003; Bowman et al., 2007; Floyd and Bowman, 2007; Rensing et al., 2008; Banks et al., 2011; Szövényi et al., 2011; Pires et al., 2013).

Despite the broad conservation of gene families across land plants, *P. amorphum* shares more orthogroups with angiosperms than it does with lycophytes or bryophytes (**Figure 3**). Nearly 5% of genes expressed in *P. amorphum* belong to orthogroups that are putatively unique to euphyllophytes (i.e., those present in ferns and seed plants, but absent in lycophytes and bryophytes). Der et al. (2011) recovered a similar pattern of shared orthogroups when considering just the gametophyte transcriptome of the fern *Pteridium aquilinum.* This greater overlap in gene family identity between ferns and seed plants likely reflects their sister relationship and the legacy of morphological innovations from their most recent common ancestor, including determinate lateral branching systems in regular, spiral phyllotaxis and increased complexity of shoot vasculature (**Figure 2**; Kenrick and Crane, 1997a; Galtier, 2010). Indeed, comparative proteomics suggests that the evolution of traits specific to euphyllophytes may have required the evolution of up to three times more new genes than the transition from gametophyte-dominant to sporophyte-dominant life cycles (Banks et al., 2011). Most notably, the greater overlap in gene family identity between ferns and angiosperms is an interesting counterpoint to our finding that *P. amorphum* has phase-specific gene expression profiles that are more like bryophytes and lycophytes rather than seed plants (see discussion below).

### Phase Specific Gene Expression in *Polypodium amorphum* Mirrors That of Gametophyte-Dominant Lineages

Perhaps the most striking result of this study is the significant overlap in the identity and expression levels of the genes expressed in fern sporophyte and gametophyte tissues. Despite having distinct global transcriptomic profiles (**Figure 5**), 97.7% of genes were expressed in both phases, with 90.2% demonstrating no statistically supported differences in expression level between the two phases. Put differently, less than 10% of the genes surveyed are responsible for the differences between the transcriptomic profiles of the phases and, potentially, the morphological and functional differences between *P. amorphum* sporophyte leaf tissue and gametophytes. Furthermore, less than 3% of genes are uniquely expressed in either the gametophyte or the sporophyte phase.

To date, expectations as to the degree of gene expression overlap between phases have been based primarily on studies of seed plants, which, despite differences in methodologies, generally suggest that the transcription profiles of gametophytes have reduced complexity and unique compositions relative to those of sporophytes. Many of the earliest studies employed a limited number of isozyme markers to compare expression between sporophyte tissues and pollen (microgametophytes) across a wide range of species, including *Lycopersicon* (Tanksley et al., 1981), *Populus* (Rajora and Zsuffa, 1986), and *Zea* (e.g., Gorla et al., 1986; Stinson et al., 1987; Willing et al., 1988). Depending on the species, between 65 and 70% of isozyme markers were expressed in both phases, but pollen always expressed fewer isozymes than sporophyte tissue. Subsequently, microarray and RNA-Seq studies investigating the embryo sac (megagametophyte) have further demonstrated that the transcription profiles of angiosperm gametophytes have a distinct and smaller set of genes relative to those of sporophytes (e.g., *Arabidopsis:* Becker et al., 2003; Honys and Twell, 2003; Pagnussat et al., 2005; Pina et al., 2005; Schmid et al., 2005; Steffen et al., 2007; Wang et al., 2010; Wuest et al., 2010; *Glycine:* Haerizadeh et al., 2009; *Oryza:* Wei et al., 2010; Ohnishi et al., 2011; *Vitis:* Fasoli et al., 2012; *Zea*: Yang et al., 2006; Davidson et al., 2011). For example, Chettoor et al. (2014) estimated that mature pollen and embryo sacs of *Zea* expressed only 53 and 88%, respectively, of the number of genes expressed in the sporophyte seedling. However, of the genes expressed in the mature pollen and embryo sacs approximately 10 and 18%, respectively, were unique to that tissue. In contrast, only 8.4% of genes expressed were specific to the *Zea* sporophyte seedling.

Several high-throughput transcriptome sequencing and microarray studies of the mosses *Funaria hygrometrica* and *P. patens* reported substantially less phase-biased gene expression than recovered in angiosperms (Szövényi et al., 2011; O’Donoghue et al., 2013; Ortiz-Ramírez et al., 2016). At one extreme, Szövényi et al. (2011) found ca. 96% overlap in the identity and expression levels of genes expressed by the two phases of *F. hygrometrica*, with less than 1% of genes uniquely expressed in the sporophyte or the gametophyte. At the other extreme, Ortiz-Ramírez et al. (2016) reported that 85% of genes are expressed by both phases of *P. patens* with as many as 10% of genes unique to the gametophyte.

A transcriptome profile study of the schizaeoid fern *L. japonicum* recovered an approximately 85% overlap in genes expressed in sporophyte and gametophyte tissues (Aya et al., 2015). While the percentage of genes expressed in both phases of *L. japonicum* is similar to our findings in *P. amorphum*, the direction of bias of phase-specific expression was the opposite: *L. japonicum* exhibited more sporophyte-specific genes, whereas *P. amorphum* exhibited more gametophyte-specific genes. Our findings in *P. amorphum* are surprising given the perceived structural and functional complexity of fern sporophytes relative to gametophytes. This may, in part, reflect our experimental design. Some genes presenting as gametophyte-specific could also be expressed in sporophyte tissues not sampled here (i.e., rhizomes or roots). Sampling additional sporophyte tissues, as well as varying growth conditions, could potentially decrease the number of gametophyte-specific genes, as well as increase the number of sporophyte-specific genes.

In general, we find that phase-biased gene expression in the polypod fern *P. amorphum* is intermediate between that reported for *Funaria* and *Physcomitrella/Lygodium*, while also being quite different from what has been recovered for angiosperms (e.g., Chettoor et al., 2014). This suggests that common life history traits may dictate the overlap in phase-specific expression, regardless of phylogenetic relatedness or life phase dominance. Despite life cycles being gametophyte-dominant in bryophytes and sporophyte-dominant in ferns (**Figure 2**), both lineages have relatively large, photosynthetic gametophytes when compared to the diminutive, non-photosynthetic gametophytes of seed plants. In addition to being photosynthetic, the gametophytes of bryophytes and most homosporous ferns are responsible for a diverse array of functions including indeterminate cell growth, cell division, rhizoids, and maintenance of existing tissue (Brandes, 1973). In contrast, the gametophytes of seed plants, especially angiosperms, are often reduced to a few cells, are embedded in and nutritionally dependent on the parent sporophyte, and have increased specificity of function primarily restricted to gamete production and/or dispersal (Friedman, 1993; Grossniklaus and Schneitz, 1998). Studies of gametophyte expression patterns in other independently evolved heterosporous lineages, such as the heterosporous water ferns (Salviniales) and heterosporous lycophytes, are necessary to discern whether endospory, the reduction in gametophyte size, and increased functional specificity result in greater distinction between phase-specific expression profiles (Bateman and DiMichele, 1994).

The extensive overlap in gene expression between sporophyte and gametophyte phases of bryophytes and polypod ferns (relative to seed plants) is in line with the antithetic hypothesis that the sporophyte evolved from a haplontic ancestor by mitotic division of the zygote (Bower, 1908; Bennici, 2008). Substantial overlap in the number and identity of genes expressed in the sporophyte and gametophyte phases of both mosses and ferns supports the hypothesis that the basic orthologous genes and gene networks necessary for sporophyte function were present in the gametophyte of the common ancestor of land plants (Pires and Dolan, 2012). Hence, the substantial overlap in phase-specific gene expression in mosses and ferns potentially reflects the ancestral condition in land plants, with the enhanced differences in phase-specific gene expression seen in angiosperms reflecting a derived condition. Differences in the phase-specific transcriptome profiles of angiosperms likely reflect the combined effects of the elaboration of the sporophyte and the extreme reduction and protection of the gametophyte.

### Phase-Specific Expression for Three Gene Families: Phototropins, Terpene Synthases, and Type II MADS-Box

Transcriptomic studies and associated GO enrichment analyses are useful for investigating the intersection of phase-specific and tissue-specific gene expression patterns with an understanding of gene function and the evolution of gene families in the broader context of land plant evolution. Studies adopting such an approach in ferns are rare. By identifying a suite of over-represented GO terms and associated genes for gametophyte and sporophyte tissues in *P. amorphum* (**Table 1** and **Supplementary Table S8**), we provide a tool for investigators interested in the regulation of genes involved in specific functional differences between fern gametophytes and sporophytes. We integrate phylogenetics and expression data from the polypod fern *P. amorphum*, as well as the schizaeoid fern *L. japonicum*, to assess phase-specific expression of three exemplar gene families associated with many highly enriched GO terms (**Supplementary Table S9**). All three have recently received significant attention for their occurrence in seed-free plants or for their importance in plant development: phototropins, terpene synthases, and Type II MADS-box genes (e.g., Li et al., 2015; Jia et al., 2016; Wilhelmsson et al., 2017).

#### Phototropins

Phototropins are photoreceptors that regulate plant physiological responses to blue light, including the accumulation of chloroplasts and phototropism (Christie, 2007). They are found in all green plants, but repeatedly underwent independent duplication events in the moss, lycophyte, fern, and seed plant lineages (Li et al., 2015). Functional analyses and knock-out studies of phototropin genes in *Arabidopsis* and *Physcomitrella* suggest that the divergent copies of phototropin have undergone sub-functionalization, with one paralog mediating chloroplast accumulation under low-light conditions (e.g., *Atphot1* in *A. thaliana*; Luesse et al., 2010) and the other mediating chloroplast avoidance under high-light conditions (e.g., *Atphot2*; Kagawa et al., 2001). In the fern *Adiantum capillus-veneris, Acphot2* has been implicated in regulating chloroplast avoidance under intense light and in cold temperatures (Kagawa et al., 2004; Kodama et al., 2008). The role of *phot1* in ferns is unknown.

Both the sporophyte and gametophyte phases of *P. amorphum* and *L. japonicum* express transcripts of fern *phot1* and fern *phot2*, but fern *phot1* is significantly up-regulated in the gametophytes of both ferns (**Figure 6**). While GO enrichment analysis suggests that fern *phot1* is broadly involved in the regulation of cellular biosynthetic and macromolecular processes (**Supplementary Tables S8, S9**), similar patterns of expression in both fern species suggest a conserved function for *phot1* among all ferns and hint at its possible role. Light availability is a limiting factor for fern gametophyte success, with an inverse relationship observed between light intensity and gametophyte mortality (Watkins et al., 2007). Furthermore, Donaher and Partanen (1971) demonstrated that relatively high intensity blue light is required for proper gametophyte development and growth in *Pteridium aquilinum*, and they hypothesized that “the ultimate form of the gametophyte is under the control of a photoreceptor that is sensitive to blue light and activated at a relatively low energy level.” These ecological and developmental studies of fern gametophytes, combined with evidence for the repeated sub-functionalization of phototropin paralogs in other plant lineages, suggest that fern *phot1* may mediate low-light physiological responses and may have an important role in facilitating proper gametophyte development under low-light conditions. Additional transcriptomic, physiological, and knock-out studies on gametophytes across the fern tree of life, will be important for determining the function of fern *phot1* in both gametophytes and sporophytes.

#### Terpene Synthases

Terpene synthases (TPS) are enzymes involved in the formation of terpenoid secondary metabolites, specialized compounds that have a myriad of roles in plant-environment interactions, such as protection against photooxidative stress and defense against herbivory (Tholl, 2006; Chen et al., 2011). In addition to plants many bacteria and fungi produce microbial terpene synthase-like (MTPSL) genes, although they differ in their primary protein structures (Li et al., 2012; Yamada et al., 2015). Jia et al. (2016) demonstrated the production of both plant-type and MTPSL genes in non-seed plants, suggesting that MTPSL genes may have been present in the common ancestor of all land plants, potentially as the result of horizontal gene transfer from a microbe or fungus, but then lost in seed-plants.

Our analysis provides strong support for the expression of both types of terpene synthases in the gametophytes and sporophytes of *P. amorphum* and *L. japonicum*, with both ferns expressing a greater diversity of MTPSL than plant-type terpene synthases (**Figure 7**). Additionally, the majority of MTPSL genes have expression that is specific-to or up-regulated in fern gametophytes, as reflected in the substantial enrichment (11-fold) of the “terpene synthase activity” GO term for genes up-regulated in the gametophyte phase (**Table 1** and **Supplementary Tables S8, S9**). This suggests they may be important for gametophyte function or defense in non-seed plants more broadly, most of which have relatively large, photosynthetic gametophyte phases. Our results are consistent with a hypothesis that MTPSL were the primary enzymes to make mono- and sesquiterpenes in early land plants (lineages with gametophyte-dominant life cycles) and that the evolution of plant-type TPS led to the eventual loss of MTPSL genes in seed plants (a lineage with highly reduced and enclosed gametophytes; Jia et al., 2016). While a few studies have demonstrated the anti-herbivory and allelopathic effects of terpenes in several fern genera such as *Pteridium* and *Adiantum* (Melos et al., 2007; Santos et al., 2010), we are the first to report phase-specific or up-regulated expression of either plant-type TPS and MTPSL genes in ferns, as well as to suggest a possible connection between the evolution of terpene synthases and the function of the gametophyte phase.

#### MADS-Box

MADS-box genes comprise an ancient family of transcription factors regulating development in animals, fungi, and plants (where they are most diverse). In plants, Type II MADS-box (MIKC) genes are strongly correlated with the origin and diversification of angiosperm reproductive structures, such as flowers and ovules (Ambrose, 2010). However, MIKC genes are also documented in gymnosperms, ferns, lycophytes, bryophytes, and streptophyte (charophyte) algae suggesting that the transcription factors regulating the development of flowers and other organs were recruited from homologous genes present in the common ancestor of nearly all lineages of green plants (Münster et al., 1997; Wilhelmsson et al., 2017). Almost all documented MIKC genes in ferns are reported from *Ophioglossum* (Münster et al., 2002), *Ceratopteris* (Kofuji and Yamaguchi, 1997; Münster et al., 1997; Hasebe et al., 1998), and *Dryopteris fragrans* (Huang et al., 2014), most of which are known to be expressed in both sporophytic and gametophytic tissues. Previous phylogenetic analyses suggest that MIKC genes in ferns and seed plants largely belong to distinct, deeply divergent clades, reflecting multiple duplications and losses of MIKC genes following the divergence of the fern and seed plant lineages ca. 466 MYA (Magallón and Hilu, 2009). One exception is the MIKC^∗^ clade, for which sequences from *Ceratopteris* are supported as sister to each of two monophyletic clades of seed plant sequences, known as the S- and P- subclades (Kwantes et al., 2011). Notably, MIKC^∗^ genes are preferentially expressed in the gametophytes of lycophytes, mosses, angiosperms, and the fern *Ceratopteris*, suggesting that they have a conserved function necessary for the development of plant gametophytes (Zobell et al., 2010; Kwantes et al., 2011).

Phylogenetic and phase-specific expression studies of MADS-box genes for the polypod fern *P. amorphum* and the schizaeoid fern *L. japonicum* greatly expand the known diversity and taxonomic representation of this gene family. Most of the *P. amorphum* and *L. japonicum* transcripts are united in four fern-specific monophyletic clades along with sequences from *Ceratopteris, Ophioglossum*, and *Dryopteris* (**Figure 8**). As with previous phylogenetic studies, these fern-specific MADS-box clades (with the exception of the MIKC^∗^ clade) are not supported as closely related to most of the 16 previously characterized angiosperm-specific MIKC genes (as summarized in Gramzow and Theissen, 2013). All but one included transcript of *P. amorphum* and *L. japonicum* are expressed in both sporophyte and gametophyte tissues (with varying degrees of preferential expression biases; **Figure 8**), which is in line with the idea that MADS-box genes have less specific roles in the control of cell differentiation and development in ferns than in seed plants (Theissen et al., 2000), and reflects their association with enriched GO terms for broadly defined cellular biosynthetic and metabolic processes (**Table 1** and **Supplementary Tables S8, S9**).

As in *Ceratopteris*, several *P. amorphum* transcripts belong to two clades that are sister to the S- and P- MIKC^∗^ subclades comprising angiosperm sequences (**Figure 8**). This sister relationship suggests an ancient gene duplication of MIKC^∗^ genes in the ancestor of ferns and seed plants. Some S- and P-type MIKC^∗^ transcripts of *Polypodium* exhibit preferential expression in gametophyte tissue (**Figure 8**). The retention of S- and P- MIKC^∗^ sequences in ferns and angiosperms, as well as their preferential expression in gametophyte tissues, lends further support for the ancient and conserved functions of both S- and P-MIKC^∗^ for regulation of gametophyte development in euphyllophytes (Kwantes et al., 2011; Liu et al., 2013). Notably, our phylogenetic analyses recovered two *P. amorphum* transcripts nested within a third MIKC^∗^ clade of sequences derived from the moss *P. patens.* These transcripts may represent a previously unreported “bryophyte-type” of MIKC^∗^ gene in ferns (Kwantes et al., 2011). Additional MADS-box sequences and phase-specific expression data from diverse ferns are necessary to test this hypothesis, and will be a boon for understanding the diversification of this important gene family across the land plant tree of life.

## Conclusion

Our study reveals substantial overlap in the identity and expression levels of transcribed genes in the gametophyte and sporophyte phases of the fern *P. amorphum*, a member of the homosporous polypod lineage that includes 80% of extant fern diversity. Our results echo those from comparable studies of mosses and lycophytes, but are distinct from studies of seed plants, in which gametophytes have expression profiles that show reduced complexity but unique composition relative to sporophytes. By contextualizing phase-specific expression in *P. amorphum* within the diversity of land plant life cycles, we conclude that plants with relatively large, photosynthetic gametophytes exhibit substantially more overlap in the identity and expression levels of genes in their gametophyte and sporophyte phases regardless of whether they have gametophyte-dominant or sporophyte-dominant life cycles. Furthermore, substantial overlap in phase-specific gene expression potentially reflects an ancestral condition in land plants, with the enhanced differences seen in angiosperms reflecting a derived condition. By integrating phylogenetic analyses with phase-specific expression information, we complement previous work on the evolution and function of phototropins, terpene synthases, and Type II MADS-box genes, with an emphasis on their putative roles in fern gametophytes.

## Author Contributions

EMS and JD designed this study, completed the data analysis, and interpreted the results. All cultivation of plant material and laboratory work was performed by EMS, with the exception of construction of the Illumina libraries which were provided by C. dePamphilis and P. Ralph, as acknowledged. The manuscript was drafted by EMS, with significant edits and intellectual contributions by ES, KP, and JD.

## Conflict of Interest Statement

The authors declare that the research was conducted in the absence of any commercial or financial relationships that could be construed as a potential conflict of interest.

## Footnotes

1 https://github.com/tanghaibao/goatools

2 www.ncbi.nlm.nih.gov

## References

[B1] Ambrose B. (2010). MADS-box genes in plant evolution and development. *Int J Plant Dev. Biol.* 4 30–37.

[B2] Andrews S. (2010). *FastQC: a Quality Control Tool for High Throughput Sequence Data.* Available at: http://www.bioinformatics.babraham.ac.uk/projects/fastqc

[B3] Aya K. Kobayashi M. Tanaka J. Ohyanagi H. Suzuki T. Yano K. (2015). De novo transcriptome assembly of a fern, *Lygodium japonicum*, and a web resource database, Ljtrans DB. *Plant Cell Physiol.* 56:e5. 10.1093/pcp/pcu184 25480117

[B4] Banks J. A. Nishiyama T. Hasebe M. Bowman J. L. Gribskov M. Albert V. A. (2011). The *Selaginella* genome identifies genetic changes associated with the evolution of vascular plants. *Science* 33 960–963. 10.1126/science.1203810 21551031 PMC3166216

[B5] Barker M. S. Wolf P. G. (2010). Unfurling fern biology in the age of genomics. *Bioscience* 60 177–185. 10.1525/bio.2010.60.3.4

[B6] Bateman R. M. DiMichele W. A. (1994). Heterospory: the most iterative key innovation in the evolutionary history of the plant kingdom. *Biol. Rev.* 69 345–417. 10.1111/j.1469-185X.1994.tb01276.x

[B7] Becker J. D. Boavida L. C. Carneiro J. Haury M. Feijo J. A. (2003). Transcriptional profiling of *Arabidopsis* tissues reveals the unique characteristics of the pollen transcriptome. *Plant Physiol.* 133 713–725. 10.1104/pp.103.028241 14500793 PMC219046

[B8] Benjamini Y. Hochberg Y. (1995). Controlling the false discovery rate: a practical and powerful approach to multiple testing. *J. R. Stat. Soc. B Methodol.* 57 289–300.

[B9] Bennici A. (2008). Origin and early evolution of land plants: problems and considerations. *Commun. Integr. Biol.* 1 212–218. 10.4161/cib.1.2.6987 19513262 PMC2686025

[B10] Berardini T. Z. Reiser L. Li D. Mezheritsky Y. Muller R. Strait E. (2015). The *Arabidopsis* information resource: making and mining the ”gold standard” annotated reference plant genome. *Genesis* 53 474–485. 10.1002/dvg.22877 26201819 PMC4545719

[B11] Bolger A. M. Lohse M. Usadel B. (2014). Trimmomatic: a flexible trimmer for Illumina sequence data. *Bioinformatics* 15 2114–2120. 10.1093/bioinformatics/btu170 24695404 PMC4103590

[B12] Borodina T. Adjaye J. Sultan M. (2011). A strand-specific library preparation protocol for RNA sequencing. *Methods Enzymol.* 500 79–98. 10.1016/B978-0-12-385118-5.00005-0 21943893

[B13] Bower F. O. (1908). *The Origin of a Land Flora: A Theory Based Upon the Facts of Alternation.* London: Macmillan Co Limited.

[B14] Bowman J. L. Floyd S. K. Sakakibara K. (2007). Green genes—comparative genomics of the green branch of life. *Cell* 129 229–234. 10.1016/j.cell.2007.04.004 17448980

[B15] Brandes H. (1973). Gametophyte development in ferns and bryophytes. *Ann. Rev. Plant Physiol.* 24 115–128. 10.1146/annurev.pp.24.060173.000555

[B16] Camacho C. Coulouris G. Avagyan V. Ma N. Papadoppoulos J. Bealer K. (2009). Blast + : architecture and applications. *BMC Bioinformatics* 10:421. 10.1186/1471-2105-10-421 20003500 PMC2803857

[B17] Chen F. Tholl D. Bohlmann J. Pichersky E. (2011). The family of terpene synthases in plants: a mid-size family of genes for specialized metabolism that is highly diversified throughout the kingdom. *Plant J.* 66 212–229. 10.1111/j.1365-313X.2011.04520.x 21443633

[B18] Chettoor A. M. Givan S. A. Cole R. A. Coker C. T. Unger-Wallace E. Vejlupkova Z. (2014). Discovery of novel transcripts and gametophytic functions in RNA-seq analysis of maize gametophyte transcriptomes. *Genome Biol.* 14 414–432. 10.1186/s13059-014-0414-2 25084966 PMC4309534

[B19] Christie J. M. (2007). Phototropin blue-light photoreceptors. *Ann. Rev. Plant Biol.* 58 21–45. 10.1146/annurev.arplant.58.032806.103951 17067285

[B20] R Core Team (2015). *R: A Language and Environment for Statistical Computing.* Vienna: R Foundation for Statistical Computing.

[B21] Davidson R. M. Hansey C. N. Gowda M. Childs K. L. Lin H. Vaillancourt B. (2011). Utility of RNA sequencing for analysis of maize reproductive transcriptomes. *Plant Genome* 4 191–203. 10.3835/plantgenome2011.05.0015

[B22] Der J. P. Barker M. S. Wickett N. J. dePamphilis C. W. Wolf P. G. (2011). De novo characterization of the gametophyte transcriptome in bracken fern, *Pteridium aquilinum*. *BMC Genomics* 12:99. 10.1186/1471-2164-12-99 21303537 PMC3042945

[B23] Donaher D. J. Partanen C. R. (1971). The role of light in the interrelated processes of morphogenesis and photosynthesis in the fern gametophyte. *Physiol. Plant.* 25 462–468. 10.1111/j.1399-3054.1971.tb01474.x

[B24] Fasoli M. Dal Santo S. Zenoni S. Tornielli G. B. Farina L. Zamboni A. (2012). The grapevine expression atlas reveals a deep transcriptome shift driving the entire plant into a maturation program. *Plant Cell* 24 3489–3505. 10.1105/tpc.112.100230 22948079 PMC3480284

[B25] Federhen S. (2012). The NCBI taxonomy database. *Nucleic Acids Res.* 40 D136–D143. 10.1093/nar/gkr1178 22139910 PMC3245000

[B26] Finn R. D. Coggill P. Eberhardt R. Y. Eddy S. R. Mistry J. Mitchell A. L. (2016). The Pfam protein families database: towards a more sustainable future. *Nucleic Acids Res.* 44 D279–D285. 10.1093/nar/gkv1344 26673716 PMC4702930

[B27] Floyd S. K. Bowman J. L. (2007). The ancestral developmental tool kit of land plants. *Int. J. Plant Sci.* 168 1–35. 10.1086/509079

[B28] Foster A. S. Gifford E. M. (1974). *Comparative Morphology of Vascular Plants* 2nd Edn. San Francisco, CA: W. H. Freeman and Company.

[B29] Frank M. H. Edwards M. B. Schultz E. R. McKain M. R. Fei Z. Sørensen I. (2015). Dissecting the molecular signatures of apical cell-type shoot meristems from two ancient land plant lineages. *New Phytol.* 207 893–904. 10.1111/nph.13407 25900772

[B30] Friedman W. E. (1993). The evolutionary history of the seed plant male gametophyte. *Trends Ecol. Evol.* 8 15–21. 10.1016/0169-5347(93)90125-9 21236093

[B31] Galtier J. (2010). The origins and early evolution of the megaphyllous leaf. *Int. J. Plant Sci.* 171 641–661. 10.1016/j.biochi.2015.03.021 25869000 PMC4678951

[B32] Gorla M. S. Frova C. Binelli G. Ottaviano E. (1986). The extent of gametophytic-sporophytic gene expression in maize. *Theor. Appl. Genet.* 72 42–47. 10.1007/BF00261452 24247769

[B33] Grabherr M. G. Haas B. J. Yassour M. Levin J. Z. Thompson D. A. Amit I. (2011). Full-length transcriptome assembly from RNA-Seq data without a reference genome. *Nat. Biotechnol.* 29 644–652. 10.1038/nbt.1883 21572440 PMC3571712

[B34] Graham L. E. Cook M. E. Busse J. S. (2000). The origin of plants: body plan changes contributing to a major evolutionary radiation. *Proc. Natl. Acad. Sci. U.S.A.* 97 4535–4540. 10.1073/pnas.97.9.4535 10781058 PMC34322

[B35] Gramzow L. Theissen G. (2013). Phylogenomics of MADS-Box genes in plants—two opposing life styles in one gene family. *Biology* 2 1150–1164. 10.3390/biology2031150 24833059 PMC3960868

[B36] Grossman J. Fernández H. Chaubey P. M. Valdés A. E. Gagliardini V. Cañal M. J. (2017). Proteogenomic analysis greatly expands the identification of proteins related to reproduction in the apogamous fern *Dryopteris affinis* ssp. *affinis*. *Front. Plant Sci.* 8:336. 10.3389/fpls.2017.00336 28382042 PMC5360702

[B37] Grossniklaus U. Schneitz K. (1998). The molecular and genetic basis of ovule and megagametophyte development. *Semin. Cell Dev. Biol.* 9 227–238. 10.1006/scdb.1997.0214 9599420

[B38] Grusz A. L. Rothfels C. J. Schuettpelz E. (2016). Transcriptome sequencing reveals genome-wide variation in molecular evolutionary rate among ferns. *BMC Genomics* 17:692. 10.1186/s12864-016-3034-2 27577050 PMC5006594

[B39] Haas B. J. Papanicolaou A. Yassour M. Grabherr M. Blood P. D. Bowden J. (2013). De novo transcript sequence reconstruction from RNA-seq using the Trinity platform for reference generation and analysis. *Nat. Protoc.* 8 1494–1512. 10.1038/nprot.2013.084 23845962 PMC3875132

[B40] Haerizadeh F. Wong C. E. Bhalla P. L. Gresshoff P. M. Singh M. B. (2009). Genomic expression profiling of mature soybean (*Glycine max*) pollen. *BMC Plant Biol.* 9:25. 10.1186/1471-2229-9-25 19265555 PMC2660330

[B41] Hasebe M. Wen C.-K. Kato M. Banks J. A. (1998). Characterization of MADS homeotic genes in the fern *Ceratopteris richardii*. *Proc. Natl. Acad. Sci. U.S.A.* 95 6222–6227. 10.1073/pnas.95.11.6222 9600946 PMC27636

[B42] Haufler C. H. Pryer K. M. Schuettpelz E. Sessa E. B. Farrar D. R. Moran R. (2016). Sex and the single gametophyte: revising the homosporous vascular plant life cycle in light of contemporary research. *BioScience* 66 928–937. 10.1093/biosci/biw108

[B43] Haufler C. H. Windham M. D. Lang F. A. Whitmore S. A. (1993). “*Polypodium*,” in *Flora of North America North of Mexico* Vol. 2 ed. >Flora of North America Editorial Committee (Oxford: Oxford University Press) 356–357.

[B44] Honys D. Twell D. (2003). Comparative analysis of the *Arabidopsis* pollen transcriptome. *Plant Physiol.* 132 640–652. 10.1104/pp.103.020925 12805594 PMC167004

[B45] Hoshizaki B. J. (1975). *Fern Growers Manual.* New York, NY: Alfred A. Knopf.

[B46] Huang Q. Li W. Fan R. Chang Y. (2014). New MADS-Box gene in fern: cloning and expression analysis of DfMADS1 from *Dryopteris fragrans*. *PLoS One* 9:e86349. 10.1371/journal.pone.0086349 24466046 PMC3899247

[B47] Iseli C. Jongeneel C. V. Bucher P. (1999). ESTScan: a program for detecting, evaluating, and reconstructing potential coding regions in EST sequences. *Proc. Int. Conf. Intell. Syst. Mol. Biol.* 7 138–148. 10786296

[B48] Jia Q. Li G. Köllner T. G. Fu J. Chen X. Xiong W. (2016). Microbial-type terpene synthase genes occur widely in nonseed land plants, but not in seed plants. *Proc. Natl. Acad. Sci. U.S.A.* 113 12328–12333. 10.1073/pnas.1607973113 27791023 PMC5087002

[B49] Kagawa T. Kasahara M. Abe T. Yoshida S. Wada M. (2004). Function analysis of phototropin2 using fern mutants deficient in blue light-induced chloroplast avoidance movement. *Plant Cell Physiol.* 45 416–426. 10.1093/pcp/pch045 15111716

[B50] Kagawa T. Sakai T. Suetsugu N. Oikawa K. Ishiguro S. Kato T. (2001). NPL1, a phototropin homologue, controls the chloroplast high-light avoidance response in *Arabidopsis*. *Science* 291 2138–2141. 10.1126/science.291.5511.2138 11251116

[B51] Kenrick P. Crane P. R. (1997a). The origin and early evolution of plants on land. *Nature* 389 33–39. 10.1038/37918

[B52] Kenrick P. Crane P. R. (1997b). *The Origin and Early Diversification of Land Plants. A Cladistic Study.* Washington, DC: Smithsonian Institution Press.

[B53] Kodama Y. Tsuboi H. Kagawa T. Wada M. (2008). Low-temperature induced chloroplast relocation mediated by a blue light receptor, phototropin 2, in fern gametophytes. *J. Plant Res.* 121 441–448. 10.1007/s10265-008-0165-9 18496648

[B54] Kofuji R. Yamaguchi K. (1997). Isolation and phylogenetic analysis of MADS genes from the fern *Ceratopteris richardii*. *J. Phytogeogr. Taxon.* 45 83–91.

[B55] Kwantes M. Liebsch D. Verelst W. (2011). How MIKC^∗^ MADS-box genes originated and evidence for their conserved function throughout the evolution of vascular plant gametophytes. *Mol. Biol. Evol.* 29 293–302. 10.1093/molbev/msr200 21813465

[B56] Lanfear R. Calcott B. Ho S. Y. W. Guindon S. (2012). PartitionFinder: combined selection of partitioning schemes and substitution models for phylogenetic analyses. *Mol. Biol. Evol.* 29 1695–1701. 10.1093/molbev/mss020 22319168

[B57] Langmead B. Trapnell C. Pop M. Salzberg S. L. (2009). Ultrafast and memory-efficient alignment of short DNA sequences to the human genome. *Genome Biol.* 10:R25. 10.1186/gb-2009-10-3-r25 19261174 PMC2690996

[B58] Li B. Dewey C. N. (2011). RSEM: accurate transcript quantification from RNA-Seq data with or without a reference genome. *BMC Bioinformatics* 12:323. 10.1186/1471-2105-12-323 21816040 PMC3163565

[B59] Li F.-W. Brouwer P. Carretero-Paulet L. Cheng S. Vries J. Delaux P.-M. (2018). Fern genomes elucidate land plant evolution and cyanobacterial symbioses. *Nat. Plants* 4 460–472. 10.1038/s41477-018-0188-8 29967517 PMC6786969

[B60] Li F.-W. Rothfels C. J. Melkonian M. Villarreal J. C. Stevenson D. W. Graham S. W. (2015). The origin and evolution of phototropins. *Front. Plant Sci.* 6:637. 10.3389/fpls.2015.00637 26322073 PMC4532919

[B61] Li G. Köllner T. G. Yin Y. Jiang Y. Chen H. Xu Y. (2012). Nonseed plant *Selaginella moellendorffii* has both seed plant and microbial types of terpene synthases. *Proc. Natl. Acad. Sci. U.S.A.* 36 14711–14715. 10.1073/pnas.1204300109 22908266 PMC3437839

[B62] Li L. Stoeckert C. J. Roos D. S. (2003). OrthoMCL: identification of ortholog groups for eukaryotic genomes. *Genome Res.* 13 2178–2189. 10.1101/gr.1224503 12952885 PMC403725

[B63] Liu Y. Cui S. Wu F. Yan S. Lin X. Du X. (2013). Functional conservation of MIKC^∗^-Type MADS box genes in *Arabidopsis* and rice pollen maturation. *Plant Cell* 25 1288–1303. 10.1105/tpc.113.110049 23613199 PMC3663268

[B64] Love M. I. Huber W. Anders S. (2014). Moderated estimation of fold change and dispersion for RNA-seq data with DESeq2. *Genome Biol.* 15:550. 10.1186/s13059-014-0550-8 25516281 PMC4302049

[B65] Luesse D. DeBlasio S. L. Hangarter R. P. (2010). Integration of phot1, phot2, and PhyB signalling in light-induced chloroplast movements. *J. Exp. Bot.* 62 4387–4397. 10.1093/jxb/erq242 20693413 PMC2955749

[B66] Maddison W. P. Maddison D. R. (2016). *Mesquite: a Modular System for Evolutionary Analysis. Version 3.10.* Availabe at: http://mesquiteproject.org

[B67] Magallón S. Hilu K. W. (2009). “Land plants (Embryophyta),” in *The Timetree of Life* eds Hedges S. B. Kumar S. (Oxford: Oxford University Press) 133–137.

[B68] Matasci N. Hung L. H. Yan Z. Carpenter E. J. Wickett N. J. Mirarab S. (2014). Data access for the 1,000 Plants (1KP) project. *GigaScience* 3:17. 10.1186/2047-217X-3-17 25625010 PMC4306014

[B69] Melos J. L. Silva L. B. Peres M. T. Mapeli A. M. Faccenda O. Anjos H. H. (2007). Chemical composition and evaluation of allelopathic potentials of *Adiantum tetraphyllum* Humb. and Bonpl. ex. Willd (Pteridaceae). *Quím. Nova* 30 292–297. 10.1590/S0100-40422007000200010

[B70] Miller J. H. Miller P. M. (1961). The effects of different light conditions and sucrose on the growth and development of the gametophyte of the fern *Onoclea sensiblis*. *Am. J. Bot.* 48 154–159. 10.2307/2439097

[B71] Münster Y. Faigl W. Saedler H. Theissen G. (2002). Evolutionary aspects of MADS-box genes in the eusporangiate fern *Ophioglossum*. *Plant Biol.* 4 474–483. 10.1055/s-2002-34130

[B72] Münster Y. Pahnke J. Di Rosa A. Kin J. T. Martin W. Saedler H. (1997). Floral homeotic genes were recruited from homologous MADS-box genes preexisting in the common ancestor of ferns and seed plants. *Proc. Natl. Acad. Sci. U.S.A.* 94 2415–2420. 10.1073/pnas.94.6.2415 9122209 PMC20102

[B73] Murray B. G. (1985). Karyotypes and nuclear DNA amounts in *Polypodium* L. (Polypodiaceae). *Bot. J. Linn. Soc.* 90 209–216. 10.1111/j.1095-8339.1985.tb00380.x

[B74] Niklas K. J. Kutschera U. (2010). The evolution of the land plant life cycle. *New Phytol.* 185 27–41. 10.1111/j.1469-8137.2009.03054.x 19863728

[B75] Nishiyama T. Fujita T. Shin T. Seki M. Nishide H. Uchiyama I. (2003). Comparative genomics of *Physcomitrella patens* gametophytic transcriptome and *Arabidopsis thaliana*: implication for land plant evolution. *Proc. Natl. Acad. Sci. U.S.A.* 100 8007–8012. 10.1073/pnas.0932694100 12808149 PMC164703

[B76] O’Donoghue M.-T. Chater C. Wallace S. Gray J. E. Beerling D. J. Fleming A. J. (2013). Genome-wide transcriptomic analysis of the sporophyte of the moss Physcomitrella patens. *J. Exp. Bot.* 12 3567–3581. 10.1093/jxb/ert190 23888066 PMC3745722

[B77] Ohnishi T. Takanashi H. Mogi M. Takahashi H. Kikuchi S. Yano K. (2011). Distinct gene expression profiles in egg and synergid cells of rice as revealed by cell type-specific microarrays. *Plant Physiol.* 155 881–891. 10.1104/pp.110.167502 21106719 PMC3032473

[B78] O’Leary N. A. Wright M. W. Brister J. R. Ciufo S. Haddad D. McVeigh R. (2016). Reference sequence (RefSeq) database at NCBI: current status, taxonomic expansion, and functional annotation. *Nucleic Acids Res.* 44 D733–D745. 10.1093/nar/gkv1189 26553804 PMC4702849

[B79] Ortiz-Ramírez C. Hernandez-Coronado M. Thamm A. Catarino B. Wang M. Dolan L. (2016). A transcriptome atlas of *Physcomitrella patens* provides insights into the evolution and development of land plants. *Mol. Plant* 9 205–220. 10.1016/j.molp.2015.12.002 26687813

[B80] Pagnussat G. C. Yu H. J. Ngo Q. A. Rajani S. Mayalagu S. Johnson C. S. (2005). Genetic and molecular identification of genes required for female gametophyte development and function in *Arabidopsis*. *Development* 132 603–614. 10.1242/dev.01595 15634699

[B81] Pedersen S. Simonsen V. Loeschcke V. (1987). Overlap of gametophytic and sporophytic gene expression in barley. *Theor. Appl. Genet.* 75 200–206. 10.1007/BF00249164

[B82] Pina C. Pinto F. Feijó J. A. Becker J. D. (2005). Gene family analysis of the *Arabidopsis* pollen transcriptome reveals biological implications for cell growth, division control, and gene expression regulation. *Plant Physiol.* 138 744–756. 10.1104/pp.104.057935 15908605 PMC1150393

[B83] Pinson J. B. Chambers S. M. Nitta J. H. Kuo L. Y. Sessa E. B. (2017). The separation of generations: biology and biogeography of long-lived sporophyteless fern gametophytes. *Int. J. Plant Sci.* 178 1–18. 10.1086/688773

[B84] Pires N. D. Dolan L. (2012). Morphological evolution in land plants: new designs with old genes. *Philos. Trans. R. Soc. Lond. B Biol. Sci.* 367 508–518. 10.1098/rstb.2011.0252 22232763 PMC3248709

[B85] Pires N. D. Yi K. Breuninger H. Catarino B. Menand B. Dolan L. (2013). Recruitment and remodeling of an ancient gene regulatory network during land plant evolution. *Proc. Natl. Acad. Sci. U.S.A.* 110 9571–9576. 10.1073/pnas.1305457110 23690618 PMC3677440

[B86] PPG I (2016). A community-derived classification for extant lycophytes and ferns. *J. Syst. Evol.* 54 563–603. 10.1111/jse.12229

[B87] Qui Y.-L. Taylor A. B. McManus H. A. (2012). Evolution of the life cycle in land plants. *J. Syst. Evol.* 50 171–194. 10.1111/j.1759-6831.2012.00188.x

[B88] Raghavan V. (2005). *Developmental Biology of Fern Gametophytes* Vol. 20 Cambridge: Cambridge University Press.

[B89] Rajora O. P. Zsuffa L. (1986). Sporophytic and gametophytic gene expression in *Populus deltoides* Marsh., *P. nigra* L., and *P. maximowiczii* Henry. *Can. J. Genet. Cytol.* 28 476–482. 10.1139/g86-071

[B90] Rambaut A. Suchard M. A. Xie D. Drummond A. J. (2014). *Tracer v1.6.* Available at: http://tree.bio.ed.ac.uk/software/tracer/

[B91] Rensing S. A. Lang D. Zimmer A. D. Terry A. Salamov A. Shapiro H. (2008). The *Physcomitrella* genome reveals evolutionary insights into the conquest of land by plants. *Science* 319 64–69. 10.1126/science.1150646 18079367

[B92] Robinson M. D. Oshlack A. (2010). A scaling normalization method for differential expression analysis of RNA-seq data. *Genome Biol.* 11:R25. 10.1186/gb-2010-11-3-r25 20196867 PMC2864565

[B93] Ronquist F. Telensko M. van der Mark P. Ayres D. L. Darling A. Hönha S. (2012). MrBayes 3.2: efficient Bayesian phylogenetic inference and model choice across a large model space. *Syst. Biol.* 61 539–542. 10.1093/sysbio/sys029 22357727 PMC3329765

[B94] Rutley N. Twell D. (2015). A decade of pollen transcriptomics. *Plant Reprod.* 28 73–89. 10.1007/s00497-015-0261-7 25761645 PMC4432081

[B95] Santos M. G. Kelecom A. de Paiva S. R. de Moraes M. G. Rocha L. Garrett R. (2010). Phytochemical studies in pteridophytes growing in Brazil: A review. *Am. J. Plant Sci. Biotechnology* 4 113–125.

[B96] Sato T. (1982). Phenology and wintering capacity of sporophytes and gametophytes of ferns native to northern Japan. *Oecologia* 55 53–61. 10.1007/BF00386718 28309902

[B97] Schmid M. Davison T. S. Henz S. R. Pape U. J. Demar M. Vingron M. (2005). A gene expression map of *Arabidopsis thaliana* development. *Nat. Genet.* 37 501–506. 10.1038/ng1543 15806101

[B98] Sessa E. B. Der J. P. (2016). “Evolutionary genomics of ferns and lycophytes,” in *Advances in Botanical Research* Vol. 78 ed. Rensing S. A. (New York, NY: Academic Press) 215–254.

[B99] Sigel E. M. Windham M. D. Haufler C. H. Pryer K. M. (2014). Phylogeny, divergence time estimates, and phylogeography of the diploid species of the *Polypodium vulgare* complex (Polypodiaceae). *Syst. Bot.* 39 1042–1055. 10.1600/036364414X683921

[B100] Simão F. A. Waterhouse R. M. Ioannidis P. Kriventseva E. V. Zdobnov E. M. (2015). BUSCO: assessing genome assembly and annotation completeness with single-copy orthologs. *Bioinformatics* 31 3210–3212. 10.1093/bioinformatics/btv351 26059717

[B101] Smith D. L. Robinson P. M. (1969). The effects of fungi on morphogenesis of gametophytes of *Polypodium vulgare* L. *New Phytol.* 68 113–122. 10.1111/j.1469-8137.1969.tb06424.x

[B102] Stamatakis A. (2006). RAxML-VI-HPC: maximum likelihood-based phylogenetic analyses with thousands of taxa and mixed models. *Bioinformatics* 21 2688–2690. 10.1093/bioinformatics/btl446 16928733

[B103] Steffen J. G. Kang I. H. Macfarlane J. Drews G. N. (2007). Identification of genes expressed in the *Arabidopsis* female gametophyte. *Plant J.* 51 281–292. 10.1111/j.1365-313X.2007.03137.x 17559508

[B104] Stinson J. R. Eisenberg A. J. Willing R. P. Pe M. E. Hanson D. D. Mascarenhas J. P. (1987). Genes expressed in the male gametophyte of flowering plants and their isolation. *Plant Physiol.* 83 442–447. 10.1104/pp.83.2.442 16665265 PMC1056377

[B105] Szövényi P. Rensing S. A. Lang D. Wray G. A. Shaw A.J. (2011). Generation-biased gene expression in a bryophyte model system. *ıMol*. *Biol. Evol.* 28 803–812. 10.1093/molbev/msq254 20855429

[B106] Tanksley S. D. Zamir D. Rick C. M. (1981). Evidence for extensive overlap of sporophytic and gametophytic gene expression in *Lycopersicon esculentum*. *Science* 213 453–455. 10.1126/science.213.4506.453 17760192

[B107] The Gene Ontology Consortium (2015). Gene ontology consortium: going forward. *Nucleic Acids Res.* 43 D1049–D1056. 10.1093/nar/gku1179 25428369 PMC4383973

[B108] The Pteridophyte Phylogeny Group (2016). A community-derived classification for extant lycophytes and ferns. *J. Syst. Evol.* 6 563–603. 10.1111/jse.12229

[B109] Theissen G. Becker A. Di Rosa A. Kanno A. Kim J. T. Münster T. (2000). A short history of MADS-box genes in plants. *Plant Mol. Biol.* 42 115–149. 10.1023/A:1006332105728 10688133

[B110] Tholl D. (2006). Terpene synthase and the regulation, diversity, and biological roles of terpene metabolism. *Curr. Opin. Plant Biol.* 9 1–8. 10.1016/j.pbi.2006.03.014 16600670

[B111] Tomei E. J. Wolniak S. M. (2016). Transcriptome analysis reveals a diverse family of kinesins essential for spermatogenesis in the fern *Marsilea*. *Cytoskeleton* 73 145–159. 10.1002/cm.21285 26887361

[B112] Wall P. K. Leebens-Mack J. Müller K. F. Altman N. S. dePamphilis C. W. (2008). PlantTribes: a gene and gene family resource for comparative genomics in plants. *Nucleic Acids Res.* 36 D970–D976. 10.1093/nar/gkm972 18073194 PMC2238917

[B113] Wang D. Zhang C. Hearn D. J. Kang I. H. Punwani J. A. Skaggs M. I. (2010). Identification of transcription-factor genes expressed in the *Arabidopsis* female gametophyte. *BMC Plant Biol.* 10:110. 10.1186/1471-2229-10-110 20550711 PMC3236301

[B114] Wang W. Z. Tong W. S. Li Y. Gao R. Zhang L. Chang Y. (2016). *De novo* transcriptome sequencing and comparative analysis of differentially expressed genes in *Dryopteris fragrans* under temperature stress. *Pak. J. Bot.* 48 885–898.

[B115] Watkins J. E. Jr. Mack M. C. Sinclair T. R. Mulkey S. S. (2007). Ecological and evolutionary consequences of desiccation tolerance in tropical fern gametophytes. *New Phytol.* 176 708–717. 10.1111/j.1469-8137.2007.02194.x 17822408

[B116] Wei L. Q. Xu W. Y. Deng Z. Y. Su Z. Xue Y. Wang T. (2010). Genome-scale analysis and comparison of gene expression profiles in developing and germinated pollen in *Oryza sativa*. *BMC Genomics* 11:338. 10.1186/1471-2164-11-338 20507633 PMC2895629

[B117] Whittier D. P. (1971). The value of ferns in an understanding of the alteration of generations. *BioScience* 21 225–227. 10.2307/1295690

[B118] Wilhelmsson P. K. I. Muhlich C. Ullrich K. K. Rensing S. A. (2017). Comprehensive genome wide classification reveals that many plant-specific transcription factors evolved in streptophyte algae. *Genome Biol. Evol.* 9 3384–3397. 10.1093/gbe/evx258 29216360 PMC5737466

[B119] Willing R. P. Bashe D. Mascarenhas J. P. (1988). An analysis of the quantity and diversity of messenger RNAs from pollen and shoots of Zea mays. *Theor. Appl. Genet.* 75 751–753. 10.1007/BF00265600

[B120] Wuest S. E. Vijverberg K. Schmidt A. Weiss M. Gheyselinck J. Lohr M. (2010). *Arabidopsis* female gametophyte gene expression map reveals similarities between plant and animal gametes. *Curr. Biol.* 20 506–512. 10.1016/j.cub.2010.01.051 20226671

[B121] Yamada Y. Kuzuyama T. Komatsu M. Shin-ya K. Omura S. Cane D. E. (2015). Terpene synthases are widely distributed in bacteria. *Proc. Natl. Acad. Sci. U.S.A.* 112 857–862. 10.1073/pnas.1422108112 25535391 PMC4311827

[B122] Yang H. Kaur N. Kiriakopolos S. McCormick S. (2006). EST generation and analyses towards identifying female gametophyte-specific genes in *Zea mays* L. *Planta* 224 1004–1014. 10.1007/s00425-006-0283-3 16718485

[B123] You C. Cui J. Wang H. Qi X. Kuo L. Y. Ma H. (2017). Conservation and divergence of small RNA pathways and microRNAs in land plants. *Genome Biol.* 18:158. 10.1186/s13059-017-1291-2 28835265 PMC5569507

[B124] Zhang Z. He Z. Xu S. Li X. Guo W. Yang Y. (2016). Transcriptome analyses provide insights into the phylogeny and adaptive evolution of the mangrove fern genus *Acrostichum*. *Sci. Rep.* 6:35634. 10.1038/srep35634 27782130 PMC5080628

[B125] Zobell O. Faigl W. Saedler H. Münster T. (2010). MIKC^∗^ MADS-box proteins: conserved regulators of the gametophytic generation of land plants. *Mol. Biol. Evol.* 27 1201–1211. 10.1093/molbev/msq005 20080864

