# Supplementary material for: Overlapping Patterns of Gene Expression Between Gametophyte and Sporophyte Phases in the Fern Polypodium amorphum (Polypodiales)
Source: Front Plant Sci. 2018 Oct 9;9:1450. doi: 10.3389/fpls.2018.01450 (PMC6190754; doi:10.3389/fpls.2018.01450)
Supplement: Supplementary file 4 [file Data_Sheet_1.PDF]

Supplementary Material

Overlapping patterns of gene expression between gametophyte and sporophyte phases in the fern *Polypodium amorphum* (Polypodiales)

Erin M. Sigel*, Eric Schuettpelz, Kathleen M. Pryer, Joshua P. Der

***Correspondence:** Corresponding Author: erinsigel@louisiana.edu

1 Supplementary Figures and Tables

1.1 Supplementary Figures

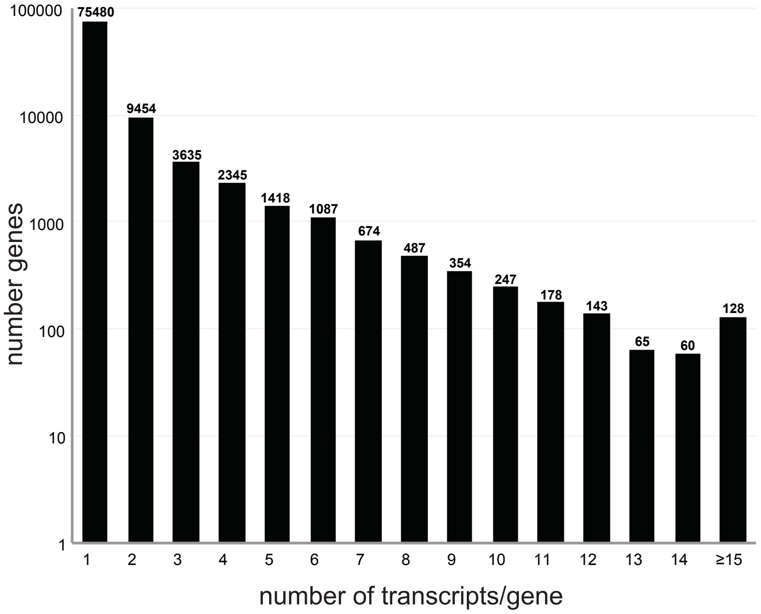

Supplementary Figure S1 Number of genes represented by one or more transcripts in the final filtered reference transcriptome of *Polypodium amorphum*. Total number of transcripts = 150,141; Total number of genes = 95,755.

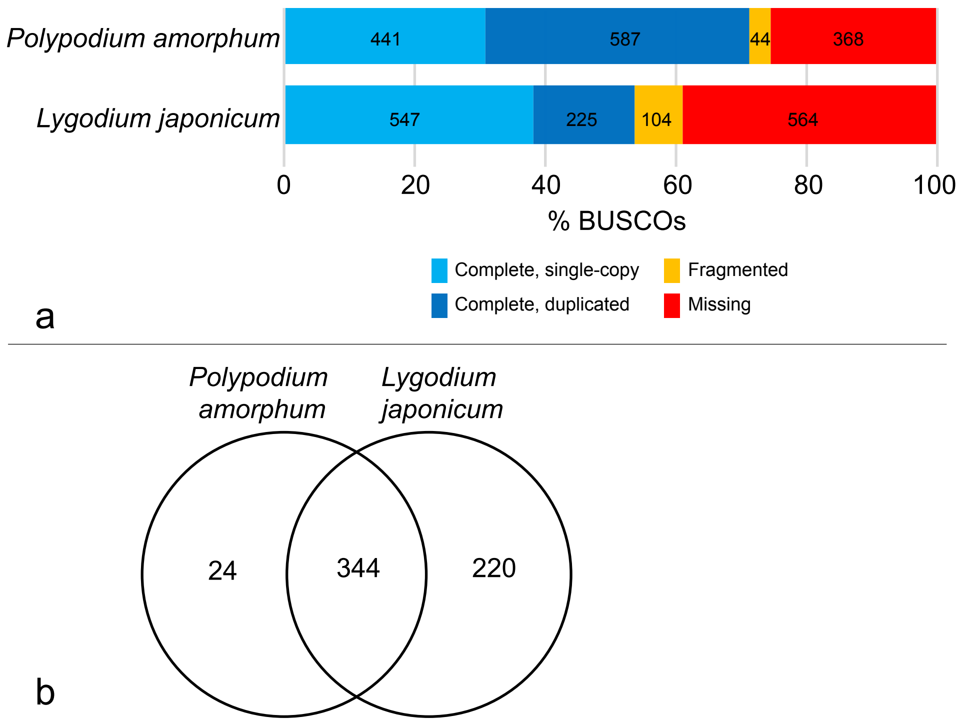

Supplementary Figure S2 Comparison of BUSCO scores (Embryophyta edb09 set) for the *Polypodium amorphum* and *Lygodium japonicum* (Aya et al., 2015) transcriptome assemblies. **a.** Blue, yellow, and red bars respectively illustrate the proportion of complete (C), fragmented (F), and missing (M) BUSCO genes; the dark blue bar is for complete but duplicated BUSCO genes. n = 1440. **b.** Comparison of missing BUSCO genes.

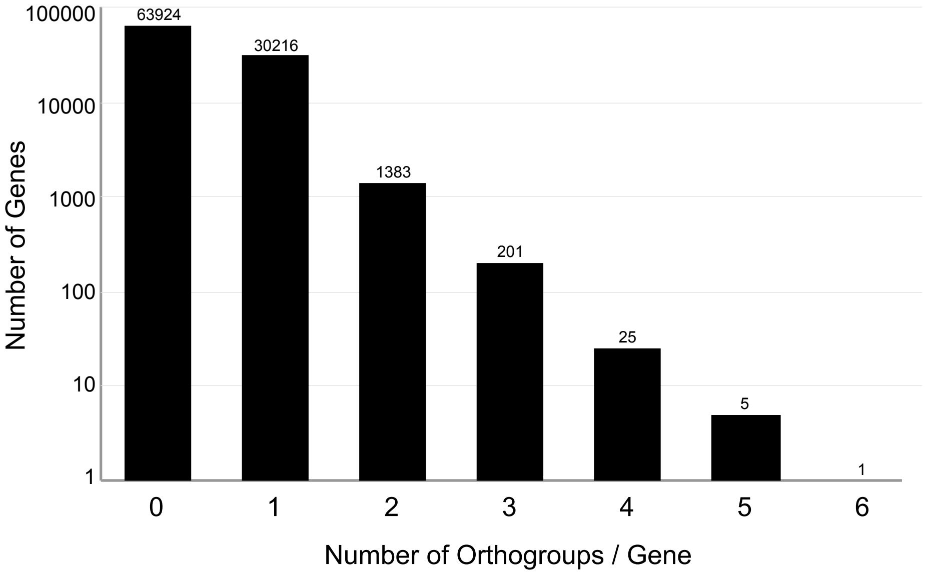

Supplementary Figure S3 Number of orthogroups assigned per gene as determined by comparison to the PlantTribes gene family classification (Wall et al., 2008). The largest group of genes, 63924, were not assigned to any orthogroup, and the next largest group of genes, 30216, were assigned to a single orthogroup.

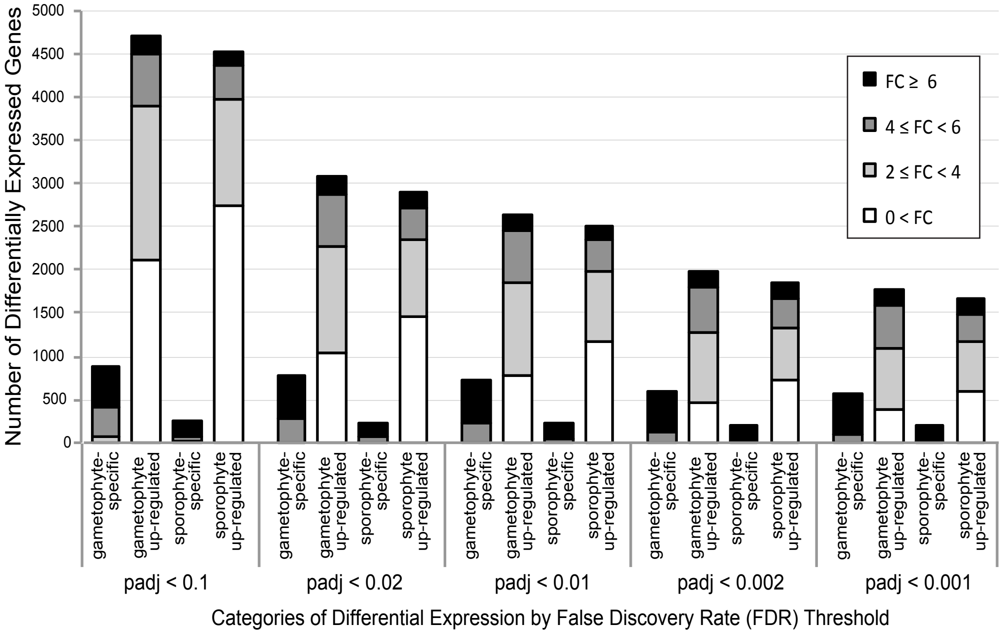

Supplementary Figure 4 Number of genes falling into four categories of differential expression—gametophyte-specific, gametophyte up-regulated, sporophyte-specific, and sporophyte up-regulated—at varying thresholds for adjusted p-values (padj) and log2-fold change (FC) in expression. As depicted in the inset, color of the bar corresponds to degree of FC.

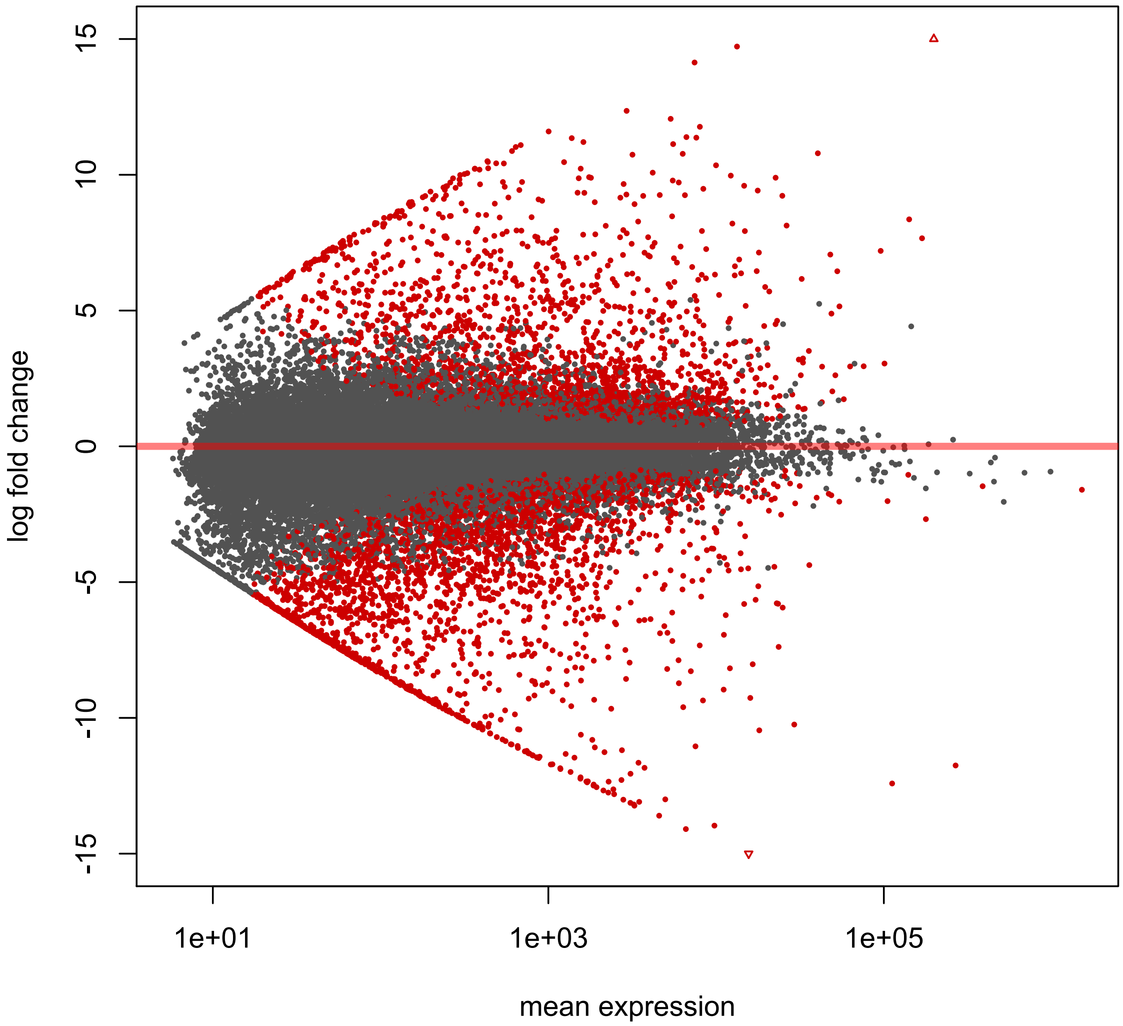

Supplementary Figure S5 MA plot of the 35169 genes included in differential expression analyses. Read count data for each of the six libraries were normalized by the corresponding size factor using DESeq2 package in R (R Development Core Team 2008; Love et al. 2014). The horizontal axis represents the average expression of a gene for all six libraries. The vertical axis represents the log2-fold change in expression between gametophyte and sporophyte libraries. Red dots indicate genes with statistically significant differences in expression between gametophyte and sporophyte libraries (log2-fold change $\geq$ 2; p-values (padj) $\leq$ 0.002), whereas grey dots indicate genes without support for differential expression.

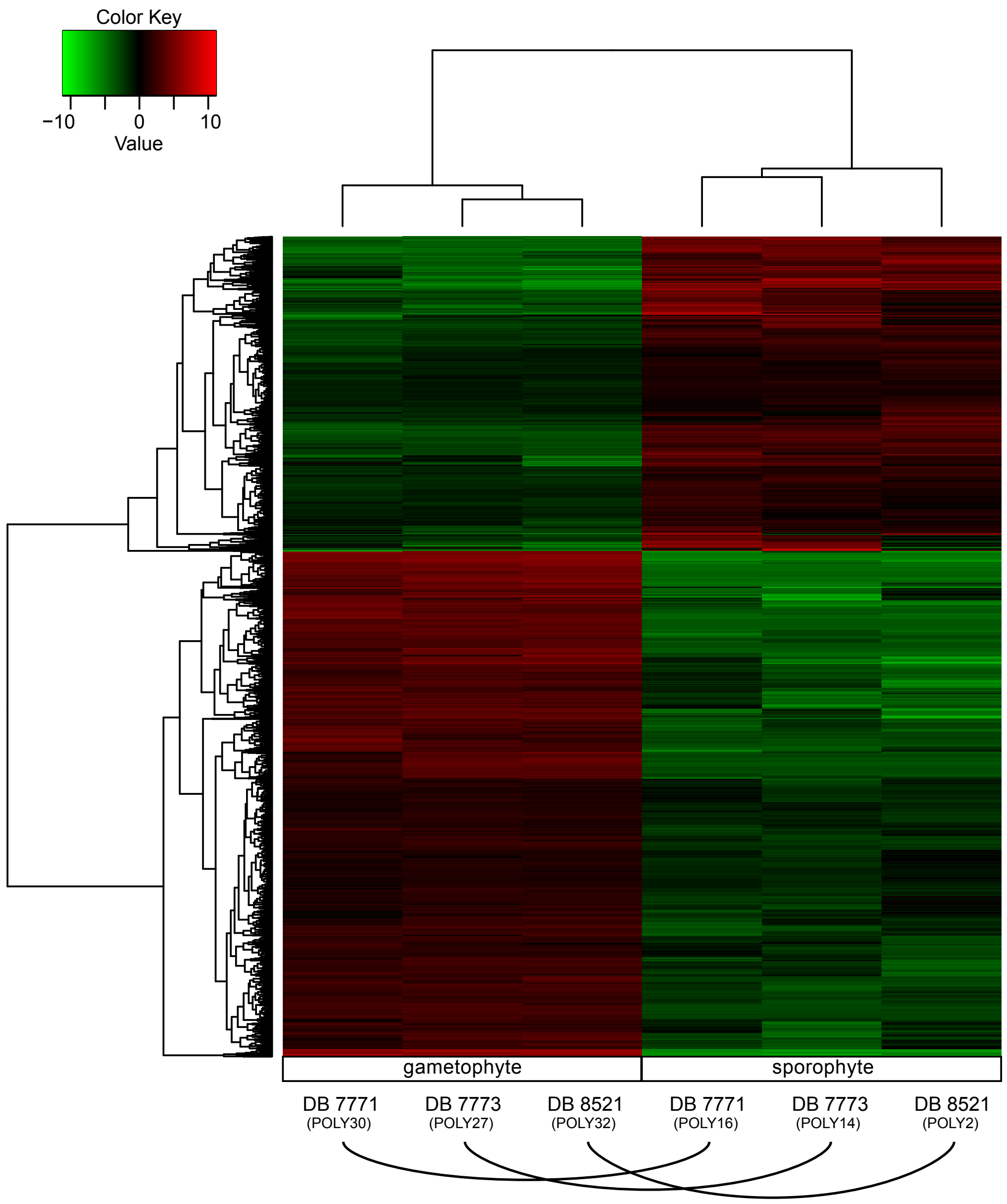

Supplementary Figure S6 Heat map of expression levels of 3447 genes exhibiting statistically significant differences in expression between gametophyte and sporophyte libraries (log2-fold change $\geq$ 2; p-values (padj) $\leq$ 0.002). Samples are labelled as gametophyte or sporophyte, with an arched line connecting samples derived from the same *P. amorphum* individual. Illumina library number for each sample is given in parentheses.

1.2 Supplementary Tables

DB Number	Sporophyte Illumina Library	Gametophyte Illumina Library	Voucher Information
7771	POLY16	POLY30	U.S.A., Washington, King County, *Sigel 2010-125*
7773	POLY14	POLY27	U.S.A., Oregon, Multnomah County, *Sigel 2010-104*
8521	POLY2	POLY32	Canada, British Columbia, Squamish-Lillooet Regional District, *Rothfels 4084*

Supplementary Table S1 *Polypodium amorphum* Suskd. specimens used in this study. DB numbers refer to unique specimen identifiers as designated in the Duke Fern Lab Database (http://fernlab.biology.duke.edu). Two Illumina cDNA libraries were generated for each specimen—one from sporophyte leaf tissue and one from whole gametophytes. All vouchers are accessioned at the DUKE herbarium.

DB Number	Illumina Library	Phase	Number of Sequenced Read Pairs	Trimmomatic Results
8521	POLY2	sporophyte	68239527	Both Surviving: 61285556 (89.81%)<br>Forward Only Surviving: 3258970 (4.78%)<br>Reverse Only Surviving: 1398881 (2.05%)<br>Dropped: 2296120 (3.36%)
7773	POLY14	sporophyte	54361763	Both Surviving: 48055949 (88.40%)<br>Forward Only Surviving: 3239533 (5.96%)<br>Reverse Only Surviving: 1050025 (1.93%)<br>Dropped: 2016256 (3.71%)
7771	POLY16	sporophyte	46369538	Both Surviving: 40775861 (87.94%)<br>Forward Only Surviving: 2807028 (6.05%)<br>Reverse Only Surviving: 937150 (2.02%)<br>Dropped: 1849499 (3.99%)
7773	POLY27	gametophyte	64843224	Both Surviving: 55822583 (86.09%)<br>Forward Only Surviving: 4178089 (6.44%)<br>Reverse Only Surviving: 1831470 (2.82%)<br>Dropped: 3011082 (4.64%)
7771	POLY30	gametophyte	63591515	Both Surviving: 54449045 (85.62%)<br>Forward Only Surviving: 4207499 (6.62%)<br>Reverse Only Surviving: 1806183 (2.84%)<br>Dropped: 3128788 (4.92%)
8521	POLY32	gametophyte	48820749	Both Surviving: 42000986 (86.03%)<br>Forward Only Surviving: 3173174 (6.50%)<br>Reverse Only Surviving: 1355698 (2.78%)<br>Dropped: 2290891 (4.69%)

Supplementary Table S3 Summary of total and trimmed number of Illumina reads for the six *Polypodium amorphum* samples included in this study. DB numbers refer to unique specimen identifiers as designated in the Duke Fern Lab Database (http://fernlab.biology.duke.edu). 100 bp paired-end reads were generated from each library.

		(A) Initial Trinity Reference Assembly	(B) Assembly Artifacts Removed (<0.01% isoforms)	(C) Non-coding Sequences Removed	(D) Duplicate Transcripts Removed	(E) Final Reference Assembly (Contaminant Transcripts Removed)
	No. transcripts	360675	280979	163827	154529	150141
	No. genes	191120	190686	98161	98161	95755
	No. assembled bases	339562154	218297584	121622346	112178607	109499757
	% GC content	44.11	44.29	47.00	46.98	47.02
Transcript Length (bases)	N50	1839	1530	1410	1392	1377
	Mean ± Std. Dev.	941.46 ± 1045.9	776.92 ± 925.37	742.38 ± 868.03	726.01± 862.51	717.88 ± 851.33
	Median	445	368	369	354	351
	Minimum	201	201	48	48	48
	Maximum	18347	18347	16560	16560	16560

Supplementary Table S4 Statistical summary of the *Polypodium amorphum* reference transcriptome for initial Trinity *de novo* assembly (A), through sequential filtering steps (B-D), to the final reference assembly (E).

	Number of Transcripts	Number of Genes
**Non-Contaminants**	64532	33128
**Contaminants**		
Bacteria	1252	1038
Fungi	641	483
Homo sapiens	11	11
Other Metazoans	898	644
Other Organisms	433	165
Viridiplantae	1059	488
Virus	94	90
**Total**	4388	2919
**"No Hit"**	85609	62627
**Grand Total**	154529	98674

Supplementary Table S5 Summary of the number of *de novo* reference transcripts and genes assigned to broad taxonomic categories by a blastp comparison to the the RefSeq non-redundant peptide database (release 09/2015; O'Leary *et al.,* 2016). A transcript/gene was identified as a non-contaminant (i.e. plant) if the best subject sequence and ≥ 50% of the best five subject sequences belonged to Viridiplantae. Transcripts/genes with hits to nr but not meeting the criteria were identified as contaminants. Transcripts/genes without hits to nr were designated as "no hit."

DB Number	Illumina Library	Phase	Number of Reads Processed	Number of Reads Mapped to the Initial Reference Transcriptome Assembly	Number of Reads Mapped to the Final Reference Transcriptome Assembly
8521	POLY2	sporophyte	62684437	Mapped: 48781074 (77.82%)<br>Failed to Map: 13903363 (22.18%)	Mapped: 30436795 (48.56%)<br>Failed to Map: 32247642 (51.44%)
7773	POLY14	sporophyte	49105974	Mapped: 30423733 (61.96%)<br>Failed to Map: 18682241 (38.04%)	Mapped: 20451476 (41.65%)<br>Failed to Map: 28654498 (58.35%)
7771	POLY16	sporophyte	41713011	Mapped: 33061661 (79.26%)<br>Failed to Map: 8651350 (20.74%)	Mapped: 21965930 (52.66%)<br>Failed to Map: 19747081 (47.34%)
7773	POLY27	gametophyte	57654053	Mapped: 47170988 (81.82%)<br>Failed to Map: 10483065 (18.18%)	Mapped: 23759854 (41.21%)<br>Failed to Map: 33894199 (58.79%)
7771	POLY30	gametophyte	56255228	Mapped: 45756025 (81.34%)<br>Failed to Map: 10499203 (18.66%)	Mapped: 26754562 (47.56%)<br>Failed to Map: 29500666 (52.44%)
8521	POLY32	gametophyte	43356684	Mapped: 34880453 (80.45%)<br>Failed to Map: 8476231 (19.55%)	Mapped: 20926368 (48.27%)<br>Failed to Map: 22430316 (51.73%)

Supplementary Table S6 Summary of trimmed Illumina reads for the six *Polypodium amorphum* samples mapped back to the initial *Polypodium* reference transcriptome assembly and to the final *Polypodium* reference transcriptome assembly (see Supplementary Table 3 columns A and E, respectively). DB numbers refer to unique specimen identifiers as designated in the Duke Fern Lab Database (http://fernlab.biology.duke.edu).
